# Clinical immunity to malaria involves epigenetic reprogramming of innate immune cells

**DOI:** 10.1093/pnasnexus/pgae325

**Published:** 2024-08-06

**Authors:** Jason Nideffer, Maureen Ty, Michele Donato, Rek John, Richard Kajubi, Xuhuai Ji, Felistas Nankya, Kenneth Musinguzi, Kathleen Dantzler Press, Nora Yang, Kylie Camanag, Bryan Greenhouse, Moses Kamya, Margaret E Feeney, Grant Dorsey, Paul J Utz, Bali Pulendran, Purvesh Khatri, Prasanna Jagannathan

**Affiliations:** Department of Medicine, Stanford University, Stanford, CA 94305, USA; Department of Medicine, Stanford University, Stanford, CA 94305, USA; Department of Medicine, Stanford University, Stanford, CA 94305, USA; Infectious Diseases Research Collaboration, Kampala, Uganda; Infectious Diseases Research Collaboration, Kampala, Uganda; Institute for Immunity, Infection, and Transplantation, Stanford University, Stanford, CA 94305, USA; Infectious Diseases Research Collaboration, Kampala, Uganda; Infectious Diseases Research Collaboration, Kampala, Uganda; Department of Medicine, Stanford University, Stanford, CA 94305, USA; Department of Medicine, Stanford University, Stanford, CA 94305, USA; Department of Medicine, Stanford University, Stanford, CA 94305, USA; Department of Medicine, University of California, San Francisco, CA 94142, USA; School of Medicine, Makerere University, Kampala, Uganda; Department of Pediatrics, University of California, San Francisco, CA 94142, USA; Department of Medicine, University of California, San Francisco, CA 94142, USA; Department of Medicine, Stanford University, Stanford, CA 94305, USA; Department of Medicine, Stanford University, Stanford, CA 94305, USA; Department of Medicine, Stanford University, Stanford, CA 94305, USA; Department of Medicine, Stanford University, Stanford, CA 94305, USA

## Abstract

The regulation of inflammation is a critical aspect of disease tolerance and naturally acquired clinical immunity to malaria. Here, we demonstrate using RNA sequencing and epigenetic landscape profiling by cytometry by time-of-flight, that the regulation of inflammatory pathways during asymptomatic parasitemia occurs downstream of pathogen sensing—at the epigenetic level. The abundance of certain epigenetic markers (methylation of H3K27 and dimethylation of arginine residues) and decreased prevalence of histone variant H3.3 correlated with suppressed cytokine responses among monocytes of Ugandan children. Such an epigenetic signature was observed across diverse immune cell populations and not only characterized active asymptomatic parasitemia but also correlated with future long-term disease tolerance and clinical immunity when observed in uninfected children. Pseudotime analyses revealed a potential trajectory of epigenetic change that correlated with a child's age and recent parasite exposure and paralleled the acquisition of clinical immunity. Thus, our data support a model whereby exposure to *Plasmodium falciparum* induces epigenetic changes that regulate excessive inflammation and contribute to naturally acquire clinical immunity to malaria.

**Significance Statement**

Clinical immunity to malaria develops gradually in children living in malaria-endemic regions, but the immunological mechanisms governing this process remain largely unresolved. In the present study, we model epigenetic changes across diverse immune cell populations and demonstrate that the relative abundance of epigenetic markers within immune cells is highly correlative with disease outcomes in *Plasmodium* infection. Moreover, these epigenetic signatures correlate with innate immune cell function and predict future protection from disease. Crucially, this study highlights the consequential nature of trained immunity in the setting of natural human infection.

## Introduction

The global disease burden of malaria is substantial. In 2022, there were an estimated 249 million cases of malaria, leading to 608,000 deaths worldwide. The majority (76%) of these deaths was in children younger than the age of 5 (1). The reason for this skewed distribution is not because older individuals have developed immunity that protects against *Plasmodium* infection (2). Instead, these older individuals more frequently have low density infections that are asymptomatic, suggesting that repeated infection by *Plasmodium* conditions both an antiparasite immune response and disease tolerance, which together promote clinical immunity to malaria (3).

In the context of malaria, disease tolerance is the ability to sustain health despite considerable parasitemia (4, 5). This is conceptually distinct but may partially rely on immunological tolerance, a state of nonreactivity toward antigens that normally would be expected to elicit an immune response (6). For example, parasite-induced production of inflammatory cytokines by myeloid and other innate immune cells has been implicated in the pathogenesis of symptomatic (e.g. febrile) and severe malaria (7). Therefore, attenuation of the innate proinflammatory response likely plays an important role in enabling asymptomatic infection. In support of this hypothesis, ex vivo transcriptional studies from malaria-naïve individuals undergoing experimental malaria infection have shown that malaria upregulates IFNγ, IL-1ß, and toll-like-receptor-mediated proinflammatory signaling and antigen presentation pathways (8, 9), with similar pathways upregulated in a study of Gabonese children with uncomplicated malaria (10). In another study, multiple and/or recent malaria episodes were associated with the upregulation of genes involved in types I and II interferon responses (11). Among Malian adults with substantial prior exposure, however, inflammatory responses were blunted (9). Furthermore, a recent report suggested that monocytes from malaria-exposed Malian adults produced lower levels of inflammatory cytokines IL-1ß, IL-6, and TNFα in response to *Plasmodium falciparum*-infected red blood cells (Pf-iRBC) compared with young Malian children (12), suggesting that modified myeloid cell responses may facilitate disease tolerance.

Regarding mechanisms by which malaria may induce innate immune cell hyporesponsiveness to stimulation, it is increasingly recognized that innate cells can adapt following exposure to various antigenic stimuli—e.g. innate immune “memory.” For example, it has long been described that lipopolysaccharide (LPS) induces in monocytes a durable state of refractoriness to subsequent LPS challenge (13). More recently, stimulation with the tuberculosis vaccine bacilli Calmette-Guérin or β-glucans increases the long-term responsiveness of monocytes and/or bone marrow-derived myeloid precursors to microbial stimuli (14, 15). This modified responsiveness is thought to be mediated by changes in chromatin accessibility and cellular metabolism (16–18), and has also been described for myeloid cells following vaccination against seasonal influenza (19), as well as NK cells following CMV infection (20). In the context of malaria, a recent study demonstrated that peripheral blood mononuclear cells (PBMCs) of susceptible children displayed an inflammatory transcriptional state prior to the transmission season (21). Other studies have suggested that malaria antigen stimulation may modify the epigenetic state of monocytes, and that these changes may affect immune responses upon subsequent challenge (12, 22, 23). However, in vivo evidence of malaria-induced epigenetic changes that modulate innate cell responsivity remains lacking.

In this study, we sought to determine whether immune cell epigenetics might contribute to clinical immunity to malaria in Ugandan children. We leveraged an unbiased, systems immunology approach using whole-blood RNA sequencing (RNA-seq), epigenetics by time of flight (EpiTOF) (24), and an in vitro functional assay to demonstrate an association between patterns of histone methylation, asymptomatic parasitemia, and suppressed cytokine responses following pathogen recognition. We also show that broad histone methylation among the immune cells of previously exposed Ugandan children predicts the acquisition of disease tolerance and clinical immunity.

## Results

### Blood transcriptomics implicate epigenetics in the acquisition of clinical immunity

**Fig. 1.**
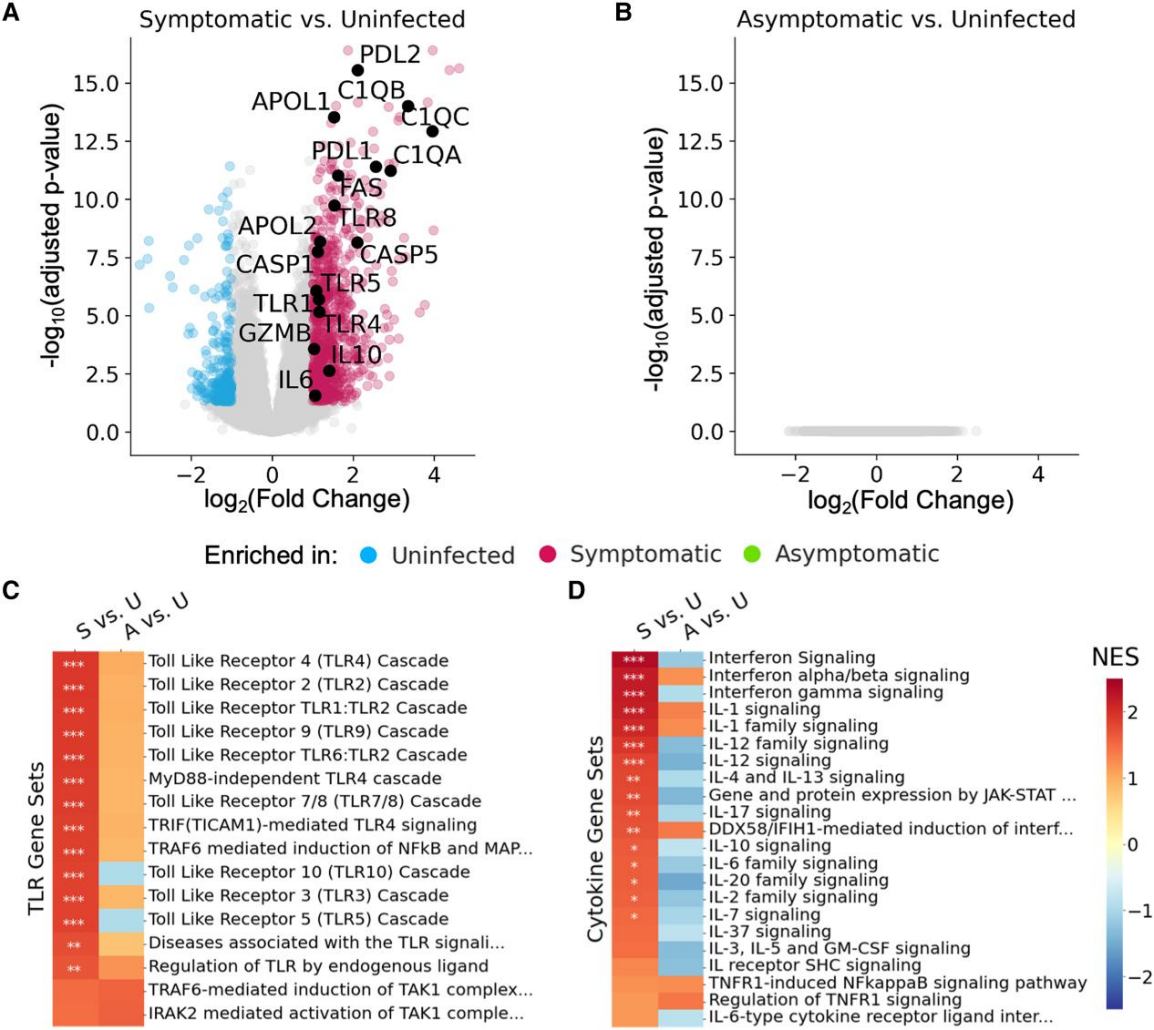
Differential gene expression in whole-blood implicates epigenetics in the outcomes of disease during *Plasmodium* infection of Ugandan children. A) Volcano plot showing differentially expressed genes in infants when they had symptomatic malaria vs. when they were uninfected. B) Volcano plot showing differentially expressed genes in children when they had asymptomatic parasitemia vs. when they were uninfected. *P*-values were adjusted to minimize false discovery. C, D) Enrichment of TLR signaling C) or cytokine-related D) gene sets in symptomatic malaria vs. uninfected (S vs. U) and asymptomatic parasitemia vs. uninfected (A vs. U). Enrichment was quantified as the normalized enrichment score (NES) between groups. Significant enrichments are annotated—*P*-value <0.05 (*); *P*-value <0.01 (**); *P*-value <0.001 (**).

We performed RNA-seq on paired, whole-blood samples from children living in malaria-endemic Uganda to determine how symptomatic malaria and asymptomatic parasitemia differentially affect the blood transcriptome compared with an uninfected timepoint (Table S1). A differential expression analysis utilizing paired samples from the same infants (*N* = 17 infants; *N* = 34 samples) identified 793 genes that were significantly upregulated and 235 genes that were significantly down-regulated during symptomatic malaria compared with an uninfected timepoint (Adjusted *P*-value <0.05, log_2_ fold change >1). Notably, complement genes (*C1QA, C1QB, C1QC*), apoptotic genes (*PDL1*, *PDL2*, *APOL1*, *APOL2*, *FAS*), inflammatory caspases (*CASP1*, *CASP5*), TLR pathway genes (*TLR1*, *TLR4*, *TLR5*, *TLR8*), cytokines (*IL6*, *IL10*), and granzyme (*GZMB*) were among the genes upregulated during symptomatic malaria (Fig. 1A). Gene set enrichment analysis (GSEA) revealed interferon signaling and antigen processing/presentation as among the top 10 gene sets upregulated in children when they had symptomatic malaria (Fig. S1A). In contrast, a separate experiment utilizing paired samples from children ages 2–12 (*N* = 14) revealed no genes upregulated or down-regulated compared with an uninfected timepoint when children had asymptomatic parasitemia (Fig. 1B). We utilized GSEA to increase our power to detect differences in transcriptional programs between asymptomatic parasitemia and an uninfected timepoint (25), and we found gene sets related to phagocytosis, B cell and FC receptor signaling, complement activation, and calcium-mediated immune cell activation were among the top 10 down-regulated gene sets in these children (Fig. S1B). Interestingly, nearly all gene sets related to TLR signaling upregulated during symptomatic malaria also trended toward upregulation during asymptomatic parasitemia (Fig. 1C), but cytokine signaling-related gene sets that were upregulated during symptomatic malaria trended toward down-regulation during asymptomatic parasitemia (Fig. 1D). Based on these data, we hypothesized that clinical immunity to malaria might be mediated by the regulation of inflammation downstream of pattern recognition, perhaps at the level of epigenetics.

### Epigenetic markers of asymptomatic parasitemia correlate with suppressed cytokine responses in monocytes

**Fig. 2.**
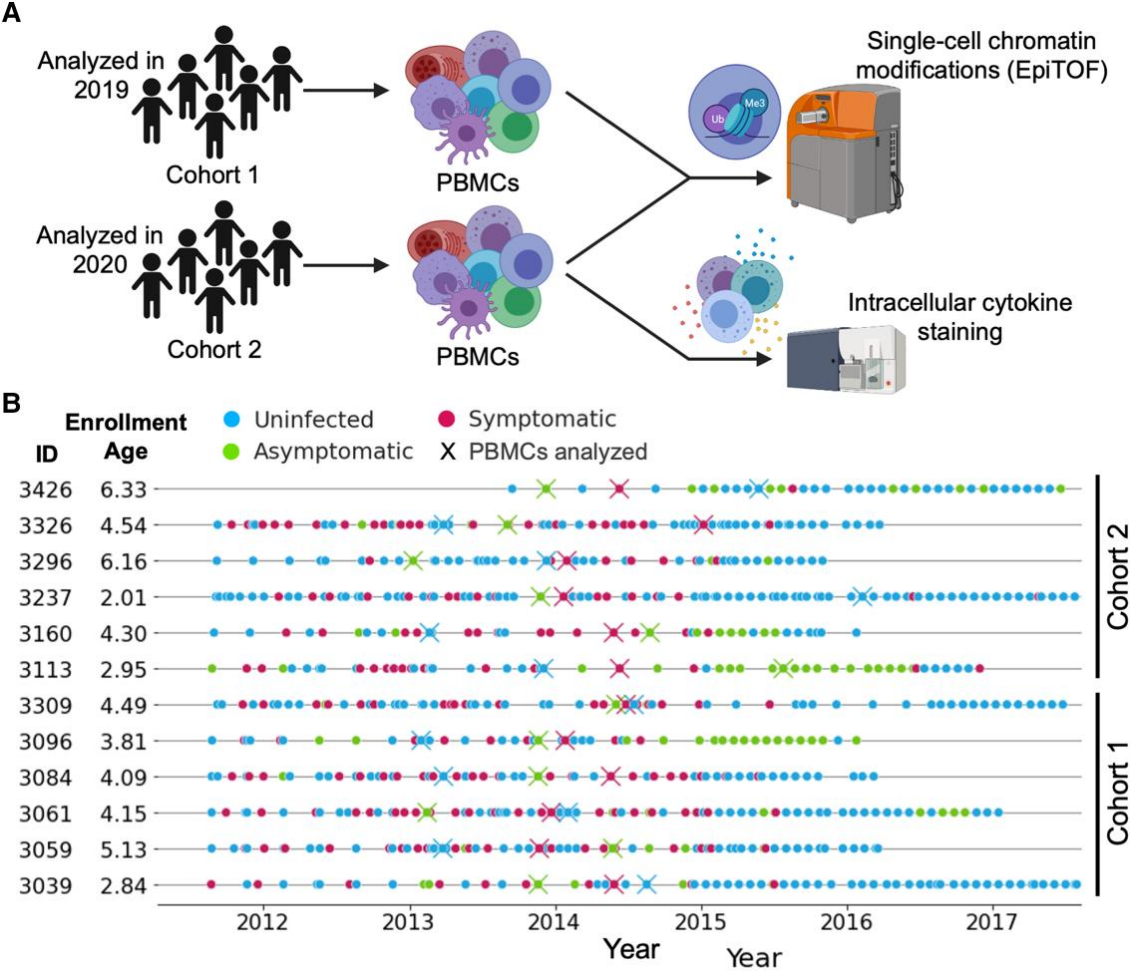
Experimental design to assess epigenetic signatures that characterize clinical immunity in children living in malaria-endemic Uganda. A) Single-cell chromatin modifications were analyzed by EpiTOF. Twelve children were sampled when they were uninfected, when they had asymptomatic parasitemia, and when they had symptomatic malaria. These children were split into two cohorts (*n* = 6), the samples of which were analyzed in two separate EpiTOF experiments performed in 2019 and 2020 for cohorts 1 and 2, respectively. Intracellular cytokine staining was also performed on the samples from cohort 2. B) Timeline showing clinic visits (circles) and sample timepoints (X) of children included in our epigenetic study. Color indicates the disease state determined by qPCR and blood smear at the time of visit.

To understand whether and how immune cell epigenetics contribute to clinical immunity, we performed epigenetic landscape profiling using EpiTOF on PBMCs isolated from children living in malaria-endemic Tororo, Uganda (Fig. 2A). The 12 children in this experiment (Table S2; separate from those studied by RNA-seq) were between the ages of 3 and 10 and had significant exposure to *P. falciparum* prior to their enrollment. Over the course of 4–7 years, these children were closely monitored for *Plasmodium* infections by repeated microscopy and loop-mediated isothermal amplication testing—*P. falciparum* was highly prevalent during the first 3 years of the study, but indoor residual spraying of insecticides (IRS) beginning in December of 2014 significantly decreased this prevalence in later years (26) (Fig. 2B). Three PBMC samples from each child were analyzed by EpiTOF—one sample from when they were uninfected, one from when they had symptomatic malaria, and one from when they presented with asymptomatic parasitemia (Fig. 2B). Importantly, the order of these timepoints with respect to disease state was varied across the children to ensure that time-related variables such as age would not confound our analyses. We assigned each of the 12 children to one of two cohorts (*n* = 6 each), the samples from which were analyzed during two separate experiments (Fig. 2A). By conducting the study in this way, we hoped to identify associations between epigenetics and clinical immunity that are robust and can be validated across separate cohorts. Reagent batch effects, procedural/instrument variability, and other sources of noise contributed to inter-cohort heterogeneity (Fig. S2). However, this variability is not problematic and actually serves to increase our confidence that our consistently observed associations may be reproduced by other research groups studying separate cohorts.

**Fig. 3.**
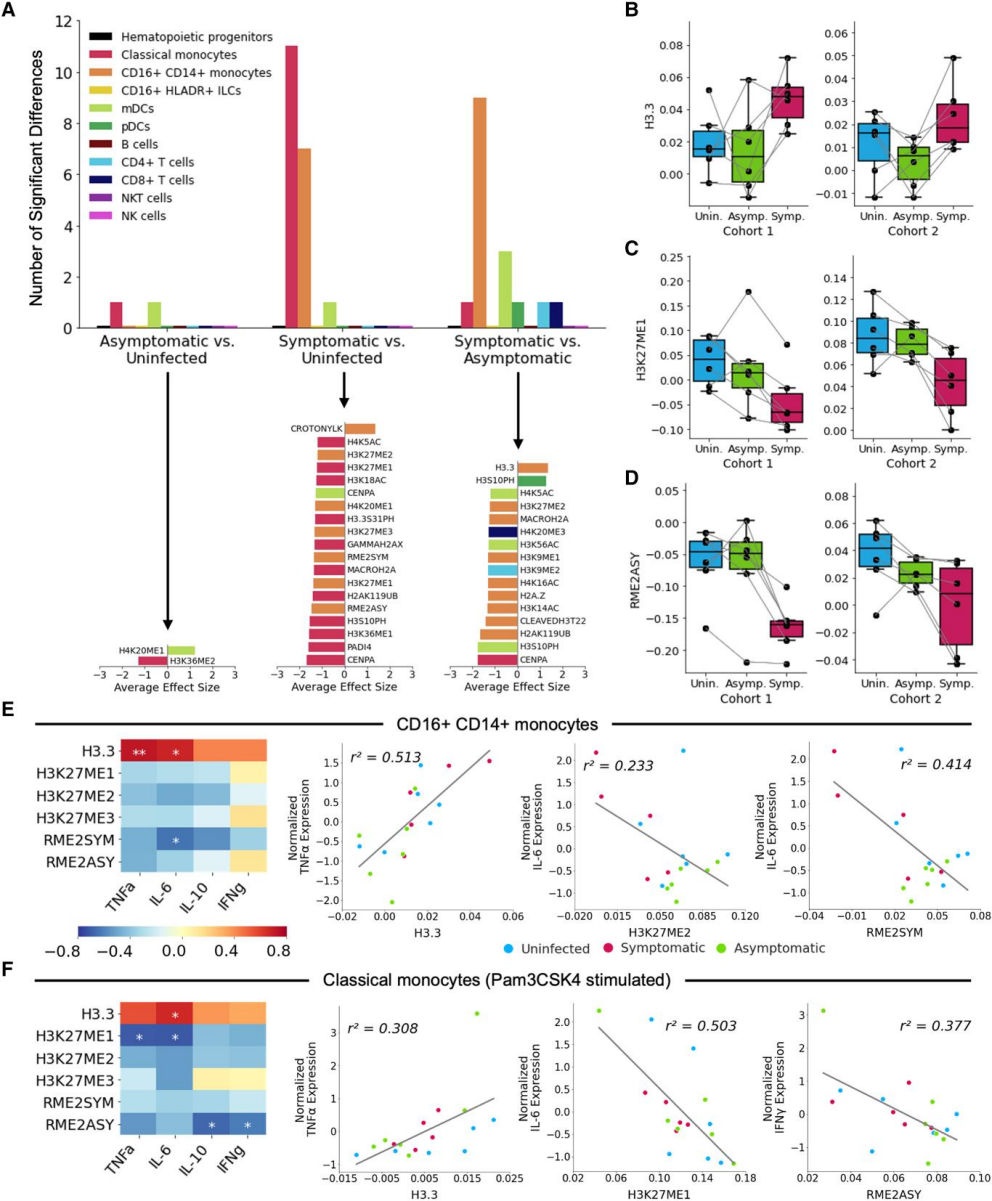
Epigenetic markers of asymptomatic parasitemia are associated with dampened cytokine responses in monocytes. A) The number (top) and effect size (bottom) of significantly differentially expressed epigenetic markers assessed via pairwise comparisons across disease states using MetaIntegrator (27, 28). Differentially expressed markers were defined as those that yielded a *P*-value <0.01 to minimize false discovery. B–D) Normalized expression of H3.3 B), *H3K27me1* (C), and *Rme2asy* (D) in CD16+ CD14^+^ monocytes across disease states. E, F) Heatmap colored by Pearson correlation coefficients describing the relationship between cytokine expression and histone marker abundance) (left side). Select regressions are shown as scatterplots (right side; colored as in B–D according to disease state). Regressions demonstrate association between histone markers and cytokine expression in unstimulated CD16^+^ monocytes E) and Pam3CSK4-stimulated classical monocytes F). *P*-value <0.05 (*); *P*-value <0.01 (**). Flow cytometric data could not be obtained for one of the symptomatic samples analyzed by EpiTOF due to a lack of cells.

Using MetaIntegrator (27, 28), a computational tool for meta-analysis, we compared the abundance of epigenetic markers in surface marker-defined immune cell populations (Fig. S3) from children sampled across disease states. In doing so, we identified two statistically significant differences between asymptomatic parasitemia and the uninfected state (Figs. 3A and S4), 19 differences between symptomatic malaria and the uninfected state (Figs. 3A and S5), and 16 differences between symptomatic malaria and asymptomatic parasitemia (Figs. 3A and S6). Classical and CD16+ monocytes were the most epigenetically distinct cell populations across disease states (Fig. 3A). Among the markers differentially abundant in CD16+ monocytes was histone H3.3, which was less abundant in asymptomatic parasitemia vs. symptomatic malaria (Fig. 3A, B). As it is relevant, H3.3 has been implicated in the positive regulation of interferon stimulated genes in mice (29). Methylation of H3K27 and H3K9, which has been associated with suppression of the interferon response (30), was generally upregulated in CD16+ monocytes of children with asymptomatic parasitemia vs. symptomatic malaria (Fig. 3A, C). Finally, arginine dimethylation (*Rme2asy*, *Rme2sym*) trended toward a greater abundance during asymptomatic infection (Fig. 3A, D). This post-translational modification, in its asymmetric form, has primarily been shown to negatively regulate inflammation (31). The differential regulation of these epigenetic markers across disease states, thus, alludes to the possibility that the epigenetic state associated with asymptomatic infection may negatively regulate cytokine production.

We next performed flow cytometry with intracellular cytokine staining to examine PBMCs from the cohort two samples for the production of IFNγ, TNFα, IL-10, and IL-6 (Figs. 2A and S7A). In doing so, we observed significant correlations between histone marker expression and basal cytokine production among CD16+ monocytes (Fig. 3E). *H3.3* was positively correlated with cytokine production, while H3K27 methylation and arginine dimethylation were negatively correlated with cytokine production (Fig. 3E). CD16+ monocytes stimulated in vitro with the TLR2/TLR1 agonist Pam3CSK4 underwent considerable cell death (Fig. S7B), confounding our ability to study cytokine production by CD16+ monocytes following stimulation of this pathway. Pam3CSK4 stimulation did not, however, deplete classical monocytes and did strongly induce their expression of TNFα and IL-6 (Fig. S7C, D). Pam3CSK4-stimulated classical monocytes demonstrated correlations between epigenetics and cytokine responses consistent with those observed in CD16+ monocytes (Fig. 3F). In sum, these data demonstrate that epigenetic patterns of asymptomatic parasitemia are associated with reduced inflammatory cytokine production by peripheral monocytes.

### Epigenetic perturbations during myelopoiesis affect monocyte function

**Fig. 4.**
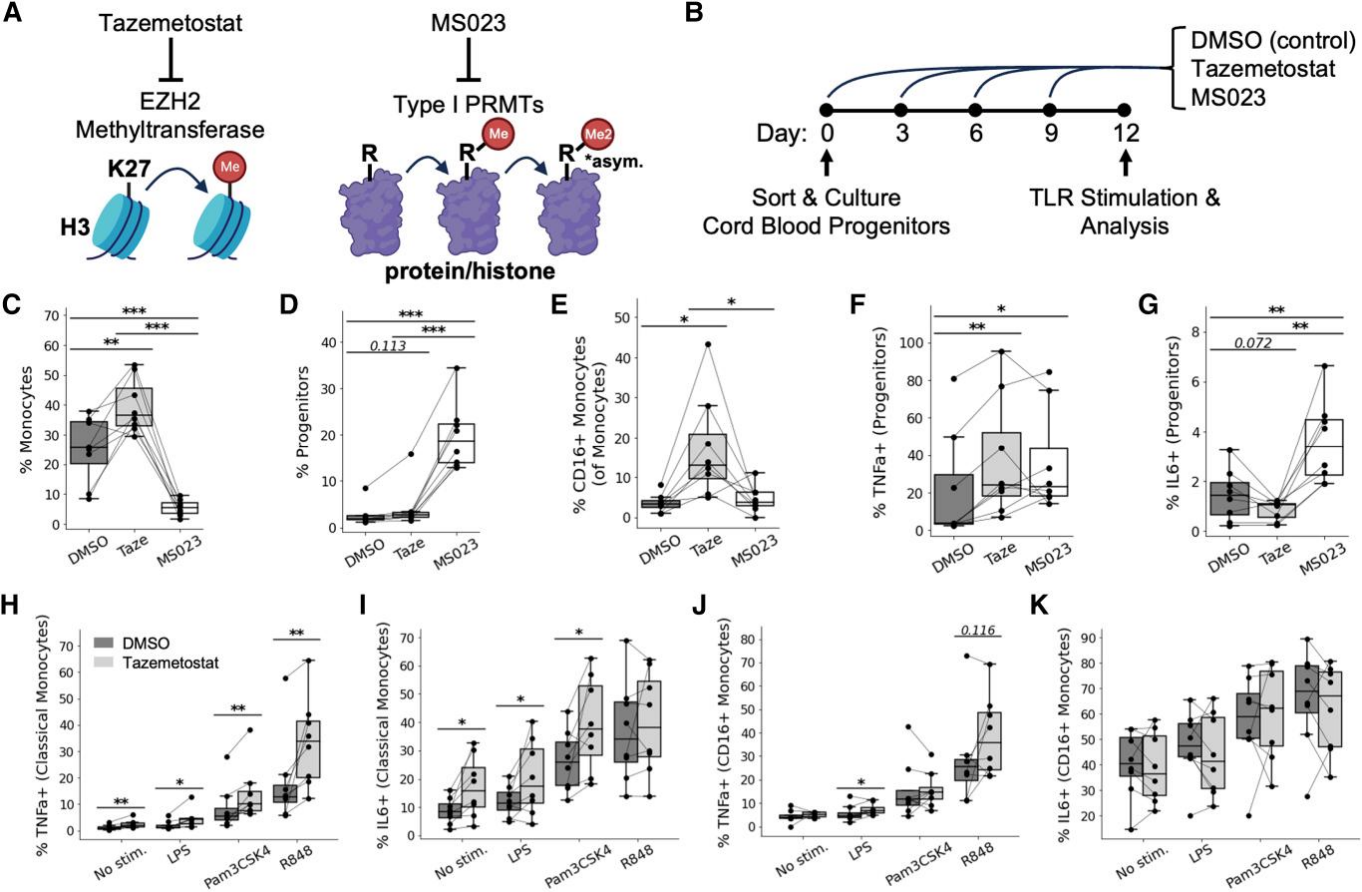
Inhibition of methyltransferases during myelopoiesis primes hematopoietic progenitors and monocytes for inflammatory cytokine production. A) Mechanisms by which tazemetostat and MS023 inhibit methyltransferases. B) Experimental design for monocyte differentiation in the presence of tazemetostat, MS023, or DMSO (control) and subsequent functional analysis. C, D) Percent of live cells that are monocytes C) or progenitors D) after differentiation. E) Percent of monocytes that are CD16^+^ after differentiation. F, G) Percent of progenitors that express TNFα (F) or IL-6 (G) after differentiation. H–K) Percent of classical monocytes. H, I) or CD16^+^ monocytes (J, K) that express TNFα (H, J) or IL-6 (I, K) in response to TLR stimulation. Statistical significance was determined by performing pairwise, paired *T* tests. *P*-value <0.05 (*); *P*-value <0.01 (**); *P*-value <0.001 (***).

To validate our observed correlations between cytokine production and epigenetics (H3K27 methylation and arginine dimethylation), we performed in vitro experiments of monocyte differentiation in the presence of methyltransferase inhibitors. Specifically, we tested whether tazemetostat (32) (which inhibits EZH2 and its ability to methylate H3K27) and MS023 (33) (which inhibits type I protein arginine methyltransferases) would alter the responsiveness of monocytes to support our observation that specific methylation patterns seen in peripheral monocytes are associated with inflammatory cytokine production (Fig. 4A). Lineage^−^ CD34^+^ hematopoietic progenitors sorted from *N* = 8 Ugandan cord blood samples were treated with methyltransferase inhibitors (or a DMSO control) every three days for the entire course of a 12-day differentiation, at the end of which, they were stimulated with TLR ligands and analyzed by flow cytometry for the production of TNFα and IL-6 (Fig. 4B). Tazemetostat and, to a much larger extent, MS023 inhibited cellular proliferation (Fig. S8A). Interestingly, MS023 significantly inhibited monocyte differentiation (Figs. 4C and S8B) and yielded a greater proportion of progenitors on day 12 (Fig. 4D). Tazemetostat, on the other hand, promoted more rapid differentiation—increasing the proportion of monocytes (Fig. 4C) and inducing substantial morphological changes by day 12 (Fig. S8C). Tazemetostat also increased the proportion of CD16+ monocytes following differentiation (Figs. 4E and S8D). Monocytes differentiated in vitro expressed HLA-DR and CD86 (Fig. S8E).

It has previously been shown that progenitors produce TNFα when exposed in vitro to IL-3 (a cytokine in our differentiation media) (34). In our study, tazemetostat and MS023 treatment both resulted in significantly increased TNFα expression among IL-3 exposed progenitors (Figs. 4F and S8F), while MS023 additionally increased IL-6 expression (Fig. 4G). Most importantly, differentiation in the presence of tazemetostat yielded classical monocytes with increased responsiveness to a host of TLR ligands: LPS (TLR4), Pam3CSK4 (TLR2/TLR1), and R848 (TLR7 and TLR8) (Fig. 4H, I). A similar trend was observed among CD16+ monocytes expressing TNFα but not IL-6 (Fig. 4J, K). In sum, these experiments demonstrate an important role for epigenetics in regulating TNFα and IL-6 production by hematopoietic progenitors and monocytes, and they support our observations in Ugandan children where lower cellular abundances of H3K27 methylation and arginine dimethylation were associated with greater inflammatory cytokine production, and vice versa.

### An immune cell-spanning epigenetic signature predicts disease tolerance and clinical immunity

**Fig. 5.**
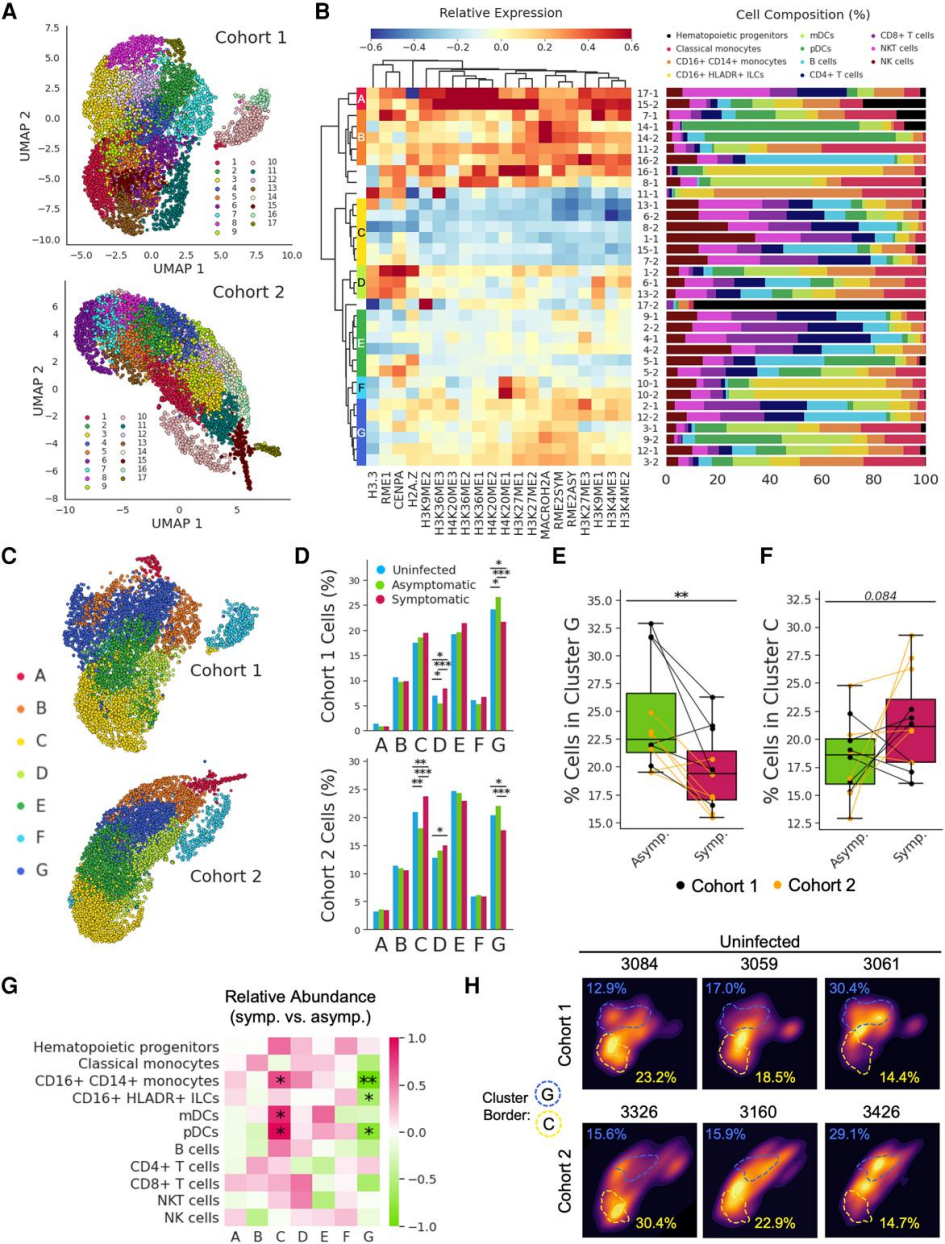
Symptomatic malaria and asymptomatic parasitemia are characterized by distinct and reproducible epigenetic signatures that span immune cell populations. A) UMAP projections and Louvain clustering performed separately on single PBMCs from children of cohort 1 and cohort 2. B) The heatmap (left) displays the relative expression of epigenetic markers (columns) across clusters (rows) from both cohorts. Original cluster identities are labeled with their cluster number and the cohort from which they were derived (i.e. cluster 17 from cohort 1 is denoted 17-1). Hierarchical clustering of the clusters yielded epigenetically related meta clusters spanning both cohorts (labeled with letters A–G). The bar graph (right) displays the percent composition of each cluster by cell type. C) Original UMAP projections of clusters 1 and 2 with cells recolored according to their meta cluster identity. The axis for the cohort 2 UMAP was rotated so that its orientation is consistent with the cohort 1 UMAP. D) Percentage of cohort 1 (top) and cohort 2 (bottom) cells from a given disease state that were assigned to each meta cluster. Statistical significance was determined using Fisher's exact test with correction for multiple hypotheses. E, F) Percentage of each child's clustered PBMCs assigned to cluster G (E) or cluster C (F). G) Relative abundance of immune cell populations assigned to specific clusters when children from cohorts 1 and 2 (analyzed together) have symptomatic malaria vs. when they have asymptomatic parasitemia. Color represents effect size calculated using Hedges’ g formula; significance was determined using a paired two-sided *T* test. H) Kernel density estimate plots depicting the distributions of cells across the UMAPs from panel “C” for six different children at the uninfected timepoint. Dashed lines show the approximate borders of clusters G and C, respectively. Similarly colored percentages denote the proportion of each uninfected child's cells that belong to the corresponding cluster. *P*-value <0.05 (*); *P*-value <0.01 (**); *P*-value <0.001 (***).

While the associations between individual epigenetic markers and disease states/cellular functions are interesting in their own right, the epigenetic landscape of a cell is governed by the combined effects of diverse post-translational modifications (35). Therefore, we performed unsupervised clustering of annotated PBMCs based on the abundance of epigenetic markers (excluding cell surface markers) in order to identify distinct epigenetic signatures spanning immune cell populations. This clustering, which was performed independently on cells down-sampled from cohorts 1 and 2, identified 17 epigenetic clusters in each cohort (Fig. 5A). Meta clustering identified conserved epigenetic clusters (A–G) across both cohorts (Fig. 5B). UMAP projections rotated and recolored according to metaclustering bore a striking resemblance (Fig. 5C), suggesting reproducible clustering. In both cohorts, cluster G was enriched in children when they had asymptomatic parasitemia vs. symptomatic malaria (Fig. 5D, E). Interestingly, this cluster was defined by low H3.3 abundance but elevated H3K27 methylation (*H3K27me1*, *H3K27me2*, *H3K27me3*) and elevated arginine dimethylation (*Rme2sym*, *Rme2asy*) (Fig. 5B), a pattern of expression that was observed in CD16+ monocytes during asymptomatic parasitemia and that was associated with diminished cytokine production (Figs. 3 and 4). Cluster C, on the other hand, trended toward enrichment in symptomatic malaria compared with asymptomatic parasitemia (Fig. 5D, F). This cluster was defined by very low methylation across several histone residues, including those that were abundantly methylated in cluster G (Fig. 5B). Importantly, these epigenetic signatures were not only associated with symptomatic malaria (cluster C) and asymptomatic parasitemia (cluster G) in a heterogeneous population of PBMCs, but they were also observed among homogenous immune cell populations of monocytes and dendritic cells from children during symptomatic malaria vs. asymptomatic parasitemia (Fig. 5G). These results demonstrate that the broad adoption of an epigenetic signature—increased methylation (especially at H3K27 and arginine residues) and decreased H3.3—by diverse immune cells is associated with asymptomatic parasitemia.

**Fig. 6.**
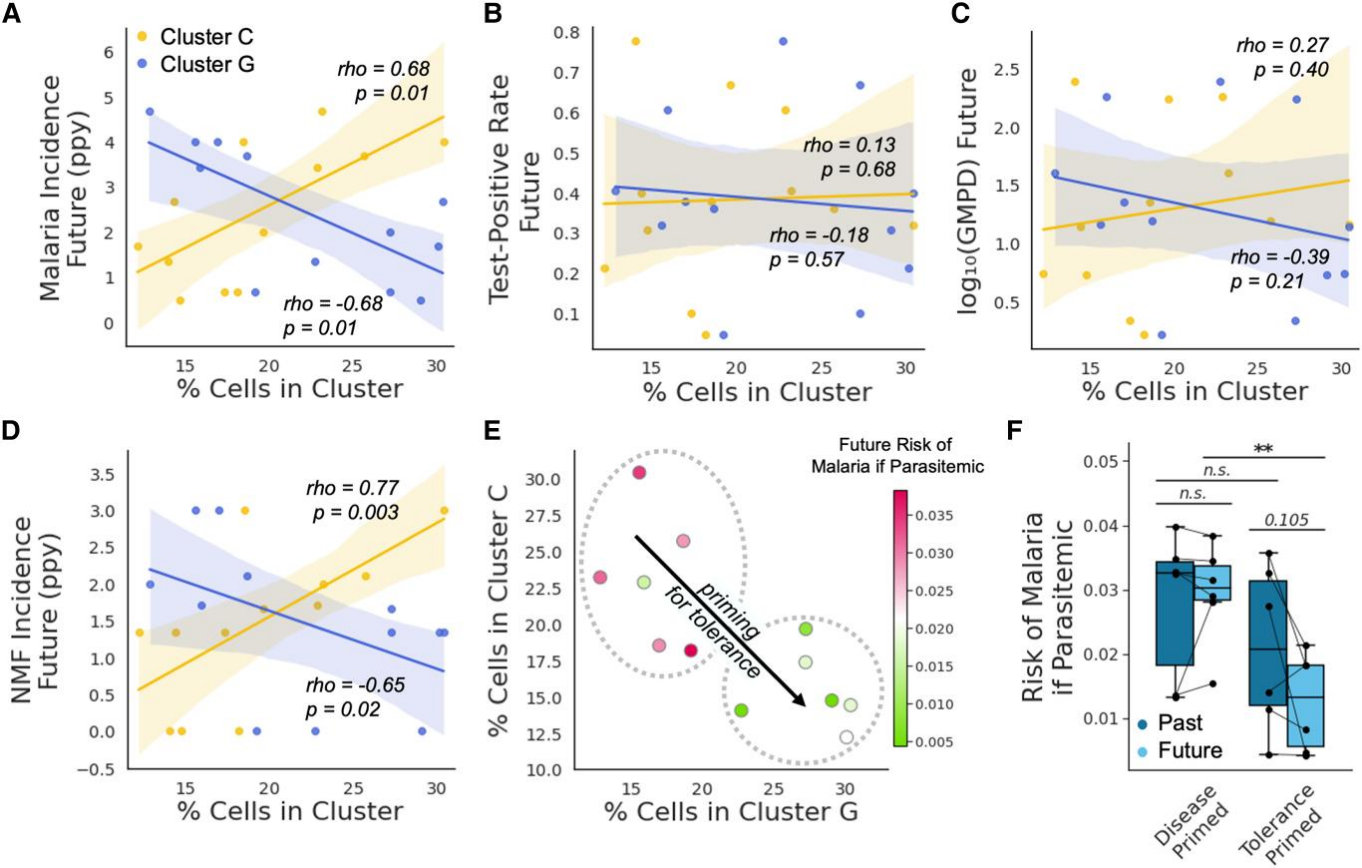
Immune cell epigenetics during homeostasis predict the acquisition of disease tolerance and clinical immunity. A–D) Scatter plots displaying the relationship between the percentage of an uninfected child's sampled PBMCs assigned to cluster G and cluster C vs. their future incidence of malaria (A), their future test-positive rate (by blood smear) (B), their future parasite burden (log-scaled geometric mean parasite density [GMPD]) (C), and their future incidence of NMF (D). Future outcome variables were calculated using data collected for duration of 3 years after the sample timepoint. Shaded regions depict 95% CIs associated with a linear regression. We also performed Spearman correlations on these data—associated *ρ* and *P* values are displayed. E) Scatter plot depicting the negative correlation between cluster G and cluster C frequencies. Each observation represents a different child, and its color denotes that child's future risk of malaria if they test parasitemia-positive. Dashed circles highlight children that are “disease primed” (upper left) or “tolerance primed” (lower right). F) Box plot depicting past and future risks of malaria given parasitemia. Children are stratified based on whether they are “disease primed” or “tolerance primed” (according to panel “E”). *P*-value < 0.01 (**).

We noticed that the distributions of cluster G and cluster C frequencies displayed greater variances among the uninfected timepoints compared with the timepoints of symptomatic malaria and asymptomatic parasitemia (Fig. 5H). The epigenetic heterogeneity observed across uninfected children, thus, led us to wonder whether a child's epigenetic state when they are uninfected primes them for a certain disease outcome upon infection. To answer this, we assessed the relationship between the frequencies of epigenetic clusters measured during an uninfected state with each child's subsequent future risk of symptomatic malaria. Indeed, the percentages of cells within cluster G and cluster C among uninfected children were negatively and positively correlated (respectively) with their future incidence of symptomatic malaria (Fig. 6A). Accordingly, the ratio of these clusters (C:G) was positively correlated with a child's future incidence of malaria (Fig. S9A). Although malaria transmission decreased significantly over the course of our study as a result of IRS (Fig. 2B), differences in the time of sampling did not confound the association between epigenetics and future risk of symptomatic malaria (Fig. S9B). The frequencies of clusters C and G did not appear to affect future risk of parasitemia (Fig. 6B) or future parasite burden (Fig. 6C), suggesting that the epigenetic state of a child's immune cells predisposes them for disease tolerance. Interestingly, cluster G and cluster C frequencies also predicted future incidences of nonmalarial fevers (NMFs) (Fig. 6D), suggesting nonspecific effects of epigenetic adaptations in these children that may have consequences beyond malaria.

Among uninfected children, cluster G and cluster C frequencies were negatively correlated (Fig. 6E). Children with high cluster G but low cluster C frequencies had a much lower future risk of malaria given parasitemia and were considered “tolerance primed” (Fig. 6E). Children with low cluster G but high cluster C frequencies were considered “disease primed.” Tolerance primed children, unlike disease primed children, had largely obtained disease tolerance and clinical immunity by the time of sampling, as they transitioned from a high-risk past to a low-risk future (Fig. 6F). We observed no correlation between the number of days since symptomatic malaria and the ratio of cluster C:G suggesting that heterogeneity in epigenetics of uninfected children is not driven by recent episodes of symptomatic malaria (Fig. S9C).

### Trajectories of epigenetic reprogramming model the gradual acquisition of clinical immunity Among Ugandan children

**Fig. 7.**
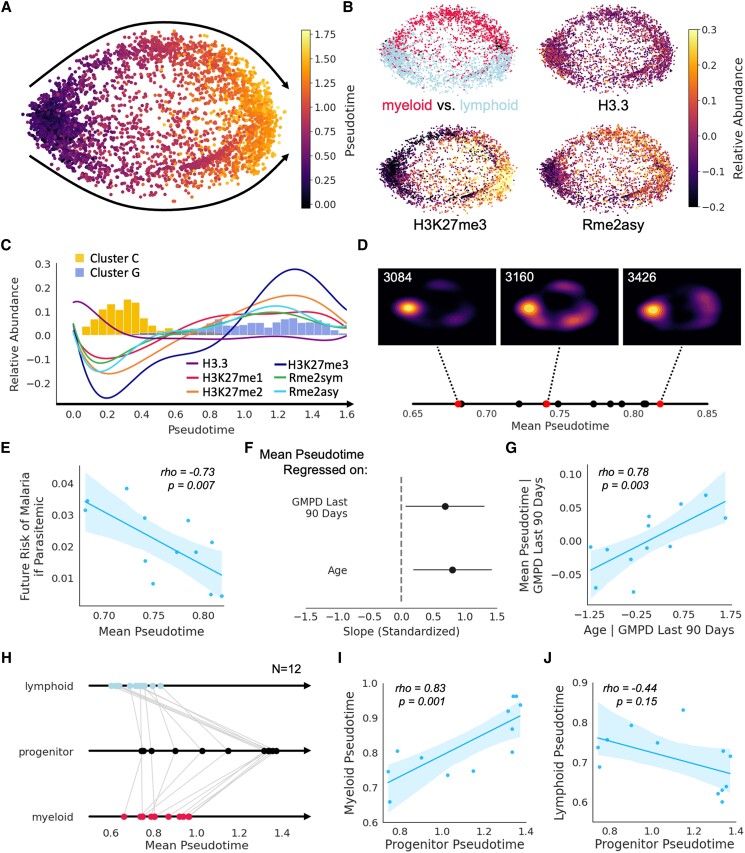
Pseudotime analyses model epigenetic changes that occur during childhood and contribute to clinical immunity among malaria-exposed Ugandans. A) tSpace projections of cells from uninfected children analyzed by EpiTOF. Cells from both cohorts (same as Fig. 4) were used and are colored based on pseudotime values. Black arrows depict two parallel trajectories through pseudotime. B) tSpace projections colored by cell lineage (top left), H3.3 abundance (top right), *H3K27me3* abundance (bottom left), or *Rme2asy* (bottom right). C) Epigenetic marker (colored lines) and cluster (colored histograms) abundance over pseudotime. D) Mean pseudotime values calculated on a per-child basis are plotted along a solid line. Kernel density estimate plots are depicted for three children with early (3084), middle (3160), and late (3426) mean pseudotime values (red dots). E) Scatter plot depicting the relationship between mean pseudotime and future risk of malaria if parasitemic, demonstrating that children who have advanced further through pseudotime are more clinically immune. F) Fitted parameter values from a multiple regression of mean pseudotime on age and recent parasite burden (measured as geometric mean parasite density [GMPD] over the preceding 90 days)—values of fitted parameters are shown with 95% CIs. Independent and dependent variables were standardized prior to model fitting. G) Scatter plot depicting a partial regression where the effect of recent parasite burden is removed and mean pseudotime is regressed on age. H) Mean pseudotime calculated for lymphoid, progenitor, and myeloid cells. Gray lines connect values calculated from cells of the same child. I, J) Scatter plots depicting the relationship between myeloid pseudotime and progenitor pseudotime I) as well as between lymphoid pseudotime and progenitor pseudotime (J). For all scatter plots, shaded regions depict 95% CIs associated with a linear regression. We also performed Spearman correlations on these data—associated *ρ* and *P*-values are displayed.

Given the inverse relationship between cluster C and cluster G among children growing up in malaria-endemic Uganda, we wondered whether a trajectory might exist whereby over the course of childhood and through repeated *Plasmodium* exposure, immune cells gradually adopt a broad methylation signature (transitioning from cluster C to cluster G). To explore this idea, we performed trajectory inference using tSpace, which seeks to uncover pathways of gradual biological change among cells projected in “trajectory space,” where cells are defined in relation to one another by their position along nearest-neighbor pathways (36). When applied to our EpiTOF data from uninfected children, this algorithm consistently yielded two trajectories (Fig. 7A). One trajectory consisted of myeloid cells and the other consisted of lymphoid cells, but both trajectories demonstrated parallel epigenetic adaptations with the passage of pseudotime (Fig. 7B). In this model, increasing pseudotime brought with it epigenetic changes associated with asymptomatic parasitemia and disease tolerance—decreasing H3.3 levels and increasing H3K27 methylation/arginine dimethylation (Fig. 7C). Additionally, passage through pseudotime demonstrated clear changes in the abundance of cluster C (which was prevalent only early in pseudotime) and cluster G (which appeared later in pseudotime) (Figs. 7C and S10).

Next, we investigated whether pseudotime in our model reflected chronological time in the lives of Ugandan children as they developed disease tolerance of malaria. To do this, we first calculated the mean pseudotime value for each of the 12 children (Fig. 7D). A strong negative correlation between the mean pseudotime value and a child's future risk of malaria if parasitemic was observed (Fig. 7E), indicating that progression through pseudotime models not only a trajectory of epigenetic reprogramming but also a trajectory through which clinical immunity is achieved. Furthermore, a child's age and recent parasite burden contributed to their progression along this trajectory (Fig. 7F, G), suggesting that the epigenetic changes observed over pseudotime model the epigenetic changes that occur as Ugandan children grow up and are repeatedly infected with *Plasmodium*. Finally, we sought to determine whether epigenetic reprogramming within the myeloid compartment might be driven by epigenetic modifications to hematopoietic stem and progenitor cells. In fact, mean pseudotime values among progenitors were highly correlated with mean pseudotime values among myeloid cells but not lymphoid cells (Fig. 7H to J), suggesting that epigenetic reprogramming may occur at the level of hematopoietic stem and progenitor cells rather than in mature myeloid cells. In sum, we have proposed a model of epigenetic reprogramming that seeks to explain the acquisition of clinical immunity through repeated exposure as children grow up in a malaria-endemic setting.

## Discussion

In this study, we utilized whole-blood transcriptomics and epigenetic profiling at single-cell resolution using EpiTOF to demonstrate that the acquisition of clinical immunity and disease tolerance is associated with epigenetic reprogramming of innate immune cells. We observed that symptomatic malaria infections were associated with upregulated interferon signaling and antigen processing/presentation genes and pathways in comparison to an uninfected timepoint, similar to prior studies conducted in malaria-naïve adults before and after controlled human malaria infection, and among symptomatic individuals living in malaria-endemic settings compared with uninfected controls (8–10). In contrast, asymptomatic infections were not associated with any significantly upregulated genes in comparison to an uninfected timepoint, recapitulating the findings of three recent studies (37–39). While it is possible that these studies were underpowered to detect such differences, they, nevertheless, demonstrate that changes in the blood transcriptome during asymptomatic parasitemia are relatively small. These observations are additionally consistent with other studies that suggested that prior malaria exposure is associated with a blunted proinflammatory transcriptional response in the setting of symptomatic infections (9, 11). Our gene set enrichment analyses identified cytokine signaling-related pathways that were upregulated during symptomatic malaria but trended toward down-regulation during asymptomatic parasitemia, suggesting that regulation of inflammation downstream of TLR signaling may contribute to clinical immunity to malaria.

These transcriptomic findings motivated an investigation into the epigenetic basis for disease tolerance of malaria. Through multiple EpiTOF experiments performed on separate cohorts, we found that decreased levels of histone H3.3 and increased arginine dimethylation and H3K27 methylation defined an epigenetic signature of innate immune cells that: (ⅰ) distinguished asymptomatic parasitemia from symptomatic malaria, (ⅱ) correlated with decreased production of cytokines, and (ⅲ) predicted the acquisition of disease tolerance in uninfected children. Further in vitro functional experiments utilizing methyltransferase inhibitors validated our observed associations between epigenetics and cytokine production in monocytes. Given these data, we hypothesize that reprogramming of innate immune cell epigenetics plays a causal role in the acquisition of disease tolerance and clinical immunity among Ugandan children repeatedly exposed to malaria.

Our data are further consistent with the hypothesis that repeated exposure to *Plasmodium* epigenetically conditions an innate immune response in favor of clinical immunity (4). We observed that the relative abundance of immune cells bearing a particular epigenetic signature correlated with future but not prior clinical immunity. The importance of temporal directionality in this association suggests that the epigenetic signature associated with disease tolerance likely emerged within the immune cells of Ugandan children over the course of our study as their cumulative parasite exposure increased. Furthermore, pseudotime analyses modeling epigenetic adaptations and the acquisition of disease tolerance revealed both age and recent parasite burden as predictors of epigenetic reprogramming.

While we suggest that an epigenetic signature associated with clinical immunity develops over time and is a deviation from the “default” state, it remains possible that variability in immune cell epigenetics across uninfected children results from epigenetic reprogramming that occurs during symptomatic episodes. If this is the case, the epigenetic state of innate immune cells may be a biomarker rather than a driver of clinical immunity in high-transmission settings. That being said, we did not see strong evidence of the persistence of a “symptomatic” epigenetic signature in the days following an episode of febrile malaria (Fig. S9C). Regardless, innate immune cell epigenetics predict clinical immunity, whether causal or correlative.

The concept of innate immune memory has been defined at the level of disease outcomes through, for example, mouse experiments demonstrating nonspecific protection against a myriad of infections following BCG vaccination (14, 15, 17, 18); however, innate immune memory has also been defined at a cellular level through in vitro experiments demonstrating a heightened or refractory response of individual cells to secondary stimulation (22). Experiments that follow in the form of the latter have clearly demonstrated that an initial stimulus can epigenetically reprogram a cell so as to alter its secondary response (12, 22). Independently, two groups recently showed that monocytes exposed to Pf-iRBCs in vitro displayed increased abundance of H3K4me3 at the TNF and IL6 loci. However, while one study demonstrated that this epigenetic modification primed monocytes for a stronger secondary response to TLR1/2 stimulation, the other study demonstrated its association with refractoriness—where monocytes produced less TNF and IL-6 upon restimulation with Pf-iRBCs. While these seemingly contradictory findings may stem from differences in the secondary stimulus, perhaps it is more likely that other experimental discrepancies between the two studies (i.e. Pf-iRBC concentration during training) differentially affected epigenetic regulators other than H3K4me3. We suspect that the abundance of just a single epigenetic marker (H3K4me3) at a few loci may not be sufficient to determine the primed state of monocytes. It is likely the case that, as our data suggests, other epigenetic regulators also exert control over innate immune memory. In our current study, we did not observe differential abundance of H3K4me3, though we did not look at specific loci. This is an important point, and it underscores the need for follow-up studies that are broad in scope, examining global abundance and locus specificity of multiple relevant epigenetic markers.

In vitro models of trained immunity may not be relevant in many in vivo contexts for the reason that innate immune cells, particularly monocytes, live a rather short life in the circulation. The phenomenon (as we have observed it in this study) that Ugandan children can exhibit disease tolerance and suppression of innate inflammatory responses after months without *Plasmodium* infection (Fig. 2B), suggests that innate immune memory is almost certainly not perpetuated by the tolerization of circulating monocytes that persist through multiple infections. Instead, we postulate that epigenetic reprogramming in favor of disease tolerance occurs at the level of stem and progenitor cells in the bone marrow and/or spleen (15). In support of this hypothesis, the epigenetic signatures that we identified to be associated with disease tolerance and intolerance delineated not only monocytes of symptomatic and asymptomatic children but also dendritic cells, B cells, and CD8^+^ T cells (Fig. 5G). The fact that similar epigenetic signatures were observed across such diverse immune cell populations and were consistent in their associations with disease tolerance suggests mutual descendance from an epigenetically reprogrammed progenitor. Furthermore, we observed a strong correlation between the epigenetic states of hematopoietic progenitors and myeloid cells derived from the same child (Fig. 7I). Together, these data support the role of progenitor cells in perpetuating innate immune memory and disease tolerance in the setting of malaria.

There were some limitations of our study. First, while we used multiple cohorts to validate our findings and utilized heterogeneity to improve generalizability, the sample size of these cohorts was relatively small. Future validations should utilize larger cohorts. Second, in our RNA-seq experiments, individuals with asymptomatic parasitemia were positive by qPCR; as a result, these analyses included smear negative, qPCR positive individuals who may have had gametocytemia and not asexual parasitemia. That being said, three recent reports also recently reported a lack of transcriptomic differences between uninfected individuals and those with asymptomatic parasitemia (37–39), consistent with our findings. Third, though we included age and recent parasite burden as independent variables in our cross-sectional models of epigenetic reprogramming, the hypothesis that repeated *Plasmodium* exposure during childhood leads to epigenetic reprogramming should be validated via longitudinal sampling of individual children at multiple timepoints across repeated infections. Fourth, while our data implicate specific epigenetic markers in disease tolerance, additional studies utilizing methods such as chromatin immunoprecipitation are needed to determine how these markers localize across the genome and affect gene expression. Finally, while our data point to epigenetically reprogrammed progenitors as the mediators of long-term tolerogenic memory it remains possible that innate immune memory is perpetuated by adaptive immune cells. For example, in a recent study, T cell-derived IFNγ was found to be required to program trained immunity in monocytes following in vitro exposure to *P. falciparum* (40). This is consistent with a separate study demonstrating that IFNγ primes chromatin to augment gene expression in response to TLR stimulation (41). In addition, IL-3 is a cytokine that enhances monocyte differentiation, potentiates cytokine storms in sepsis (42), and contributes to poor disease outcomes associated with blood infections, including malaria (42, 43). Activated T cells and B cells are a primary source of IL-3 in humans (42, 43); and therefore, it remains possible that repeated malaria could condition antigen-specific adaptive immune cells to produce less IL-3 upon activation, leading to less potent activation/priming of innate immune cell responses.

In conclusion, this work demonstrates that the relative abundance of epigenetic markers within immune cells is highly correlative with disease outcomes in *Plasmodium* infection. Moreover, the characteristic epigenetic signatures that we identify do not merely result from either asymptomatic parasitemia or symptomatic malaria; rather, these signatures predict future disease outcomes upon infection. Furthermore, key markers that defined the epigenetic signature of children primed for tolerance were associated with diminished cytokine responses in peripheral monocytes, demonstrating functional consequences of epigenetic reprogramming. Finally, by connecting age and parasite burden with epigenetics, this study proposes a model to partially explain the acquisition of clinical immunity among Ugandan children through repeated exposed to *Plasmodium*.

## Materials and methods

### Clinical study cohorts

Samples were obtained from children enrolled in longitudinal studies in Eastern Uganda. This included children living in Tororo District followed in two cohorts through the East African International Centres of Excellence in Malaria (PRISM1 [2011–2017] and PRISM 2 [2017–2019] research cohorts) (26, 44), and a third cohort of children followed from birth in neighboring Busia District (PROMOTE Birth Cohort 3 [2016–2018]) (45). In both settings, malaria transmission is high and perennial. In Tororo District, IRS with the carbamate bendiocarb was initiated in December 2014, with additional rounds administered in June 2015 and November 2015. In June 2016, IRS was administered with the organophosphate pirimiphos-methyl (Actellic), with repeated rounds in June 2017 (26). IRS has not been implemented in Busia district.

Routine assessments were performed in the study clinic every month, including blood smears and dry blood spots to detect for parasite infection. Detection methods of parasitaemia at routine visits differed in the study cohorts. In the Tororo cohorts, parasitemia was detected by microscopy (PRISM1) or an ultrasensitive qPCR assay (46, 47) (PRISM2). In the Busia birth cohort, parasite detection was performed by microscopy only. Blood samples were obtained at select visits. Further details of the cohort studies, including the defining characteristics of symptomatic malaria vs. asymptomatic parasitemia are described in the supporting methods.

Written informed consent was obtained from the parent or guardian of all study participants. The study protocols were approved by the Uganda National Council of Science and Technology, the Makerere University School of Medicine Research and Ethics Committee, the University of California, San Francisco Committee on Human Research, and the Institutional Review Boards at Stanford University.

### Whole-blood RNA-seq

Whole blood collected in RNA protect was cryopreserved before shipment to Stanford University. For RNA extraction, the mixes of whole-blood cells and RNAprotect Cell Reagents were thawed then centrifuged for 5 min at 5,000*×g*. A 350 μL QIAGEN Buffer RLT plus was added to dissolve the pellets. The QIAGEN RNeasy Plus mini (QIAGEN, Cat. #74134) kit was used for RNA purification on QIAcube automation (QIAGEN, Cat. # 9002864). RNA samples were eluted in 30 μL RNase-free water. All RNAs were checked on a NANODRP1000 and Agilent bioanalyzer 2100 RNA NANO analysis for RNA yield, purity, and integrity. The KAPA mRNA HyperPrep Kits (KK8580) with the IDT for Illumina Dual Index Adapter kit (Cat. #20021454) were used for library preparation per the manufacturer's protocol. Extra hemoglobin RNA removal and rRNA depletion using the Kapa specific reagents (RiboErase Globin) were added to the protocol. Briefly, mRNA was captured using magnetic oligo-dT beads then RiboErase Globin capture beads were applied. Fragmentation was performed using heat and magnesium. First strand cDNA synthesis was completed using random priming. Combined second strand synthesis and A-tailing, adapter ligation, library amplification, and KAPA Pure Beads clean-ups were performed for library preparation. The strand marked with dUTP is not amplified, allowing strand-specific sequencing. All final libraries were checked on Agilent's bioanalyzer 2100 High Sensitivity DNA Chip. An equal amount of cDNA library from each sample was pooled for sequencing on the Illumina Hiseq 4000 platform. FASTQ files were generated using the bcl2fastq2 Conversion v2.19 tool.

### EpiTOF

EpiTOF was performed as previously described (24). More details specific to our study are provided in the supporting methods.

### Flow cytometric analysis of pediatric samples

Cryopreserved PBMC samples were thawed in R10 media (RPMI-1640, 10% fetal bovine serum, 1 mM L-glutamine, 10 mM HEPES, spiked with pen/strep) and stimulated with Pam3CSK4 or allowed to rest for 5 h. BD GolgiPlug (Brefeldin A) was added after 1.5 h of incubation/stimulation according to the manufacturer's instructions. After incubation/stimulation, cells were stained for surface markers (CD19, CD3, HLA-DR, CD14, CD16, CD11c, and CD123). After surface staining, cells were fixed and permeabilized using the kit supplied by ThermoFisher according to the manufacturer's directions. Intracellular cytokine staining was performed using antibodies specific for IFNγ, TNFα, IL-10, and IL-6. Finally, single cells were analyzed for fluorescence using an Attune NxT flow cytometer. Gating of immune cell populations was performed as shown in Fig. S7.

### Monocyte differentiation and methyltransferase inhibition analysis

Hematopoietic progenitor cells were sorted from cord blood samples of Ugandan newborns as Lin^−^ (CD3, CD19, CD14, CD16, CD56), CD34^+^, and CD45 dim. We opted to use cord blood from Ugandans rather than North Americans, as the former population is at risk for malaria and thus more relevant to the present study. A total of 30,000 progenitors were sorted per sample and split evenly across the three culture conditions (DMSO, tazemetostat, MS023) in a 24-well plate. Culture media consisted of StemSpan SFEM Hematopoietic Cell Culture Media with Penicillin-Streptomycin and the following cytokines: IL-3 (30 ng/mL), M-CSF (30 ng/mL), Flt-3 ligand (30 ng/mL), and SCF (25 ng/mL), as previously described (48). Cells were split or provided with fresh media every 3 days. After 12 days in culture, cells were harvested and stimulated with TLR ligands (10μg/mL Pam3CSK4, 10 ng/mL LPS, 5μg/mL R848, or media control) for 5 h. BD GolgiPlug (Brefeldin A) was added after 1.5 h of incubation/stimulation to allow for intracellular cytokine staining. After incubation/stimulation, cells were stained for surface markers (HLA-DR, CD123, CD3, CD19, CD14, CD16, CD86, and CD34) and LIVE/DEAD Fixable Aqua, and then, the cells were fixed, permeabilized, and stained for intracellular cytokines (IL-6, TNF). Cells were then analyzed using an Attune NxT flow cytometer.

### Computational and statistical methods

#### Analysis of gene expression data

The STAR aligner tool was used to trim reads and align them to the reference genome (GRCh38), generating a gene expression count matrix. Differential expression of genes across disease states was analyzed using DeSeq2. This was a paired analysis, since samples from the same individuals were collected during multiple disease states. Significant differentially expressed genes were those that had an adjusted *P*-value <0.05 and absolute log_2_ fold change ≥1. Gene lists ranked according to fold-change sign and *P*-value were used to perform GSEA using the R package “fgsea” with Reactome gene sets from c2.cp.reactome.v7.0.symbols.gmt with a minimum of 15 genes and a maximum of 500 genes. Gene sets enriched in children when they had asymptomatic parasitemia vs. when they were uninfected (and vice versa) were then assessed for their enrichment in children when they had symptomatic malaria vs. when they were uninfected.

#### Supervised analysis of EpiTOF and flow cytometry data

For EpiTOF, raw data were preprocessed using FlowJo (FlowJo, LLC) to identify cell events from individual samples by palladium-based mass tags, and to segregate specific immune cell populations by immunophenotypic markers. A detailed gating hierarchy is described in Fig. S3. Single-cell data for various immune cell subtypes from individual subjects were exported from FlowJo for downstream computational analyses.

The exported Flowjo data were then normalized following previously reported methods (24). In brief, the value of each histone mark was regressed against the total amount of histones, represented by measured values of H3 and H4. Instead of co-normalizing EpiTOF data from both cohorts, we treated both cohorts as independent datasets when calculating effect sizes. We then performed a random effects inverse variance meta-analysis to combine effect sizes across datasets using MetaIntegrator (27, 28). An alpha of 0.01 was used as a threshold of significance to minimize false discovery for comparisons of marker abundance across disease states. Correction for multiple hypotheses was not performed in this case because of concerns regarding the independence of test statistics.

From our flow cytometric data, geometric means of fluorescence intensity (MFI) were calculated for each cytokine and cell population on a per-sample basis. MFI values for stimulated conditions were background subtracted. Linear regression between histone marker and cytokine expression (*z*-scored) were performed at the sample-level, and Pearson correlation coefficients for these regressions were visualized with heatmaps (with statistical significance annotated). *R*^2^ values were reported for select regressions of interest.

#### Analysis of methyltransferase inhibition experiments

For comparisons between all three treatment groups, a repeated measure ANOVA was performed followed by pairwise, paired *T* tests corrected using the Benjamini–Hochberg procedure. Otherwise, comparisons between just the DMSO and tazmetostat groups were performed using paired *T* tests.

#### Unsupervised clustering of EpiTOF data

For unsupervised analyses, weighted, random sampling of cells was performed to obtain nearly equal numbers of a given cell type across children, across disease states, and across cohorts. This ensured that differences between samples, disease states, and cohorts were a result of epigenetic changes and not changes in the proportions of surface marker-defined cell populations. UMAP projections and Louvain clustering were performed independently for cohorts 1 and 2 to identify communities of epigenetically related cells. Medians of expression for the 20 markers for each cluster were normalized to those of the other clusters in the same cohort, and then, the Euclidean distance between all clusters was calculated to construct a hierarchy using Ward's method and identify metaclusters. Metaclustering was performed using a single cutoff value that was selected because it minimizes heterogeneity within metaclusters (grouping related clusters together) while maximizing heterogeneity between metaclusters (separating clusters that are very different). Metaclusters required at least one cluster from each cohort.

#### Pseudotime analysis of EpiTOF data

Tspace was used to perform trajectory inference with the same data used in our other unsupervised analyses. Standard algorithm parameters were used as recommended by the developers. The expression of epigenetic markers over pseudotime was fit using an eight-degree polynomial function (Fig. 7C). Ordinary least squares regressions between pseudotime and clinical variables (in Fig. 7E, I, J) were performed to fit the data and display 95% confidence intervals. For these relationships, we also performed Spearman correlations and reported the associate *ρ* values and *P*-values. In Fig. 7F, a multiple linear regression was performed with standardized variables: “Age” and “GMPD Last 90 Days,” as independent variables and mean pseudotime as a dependent variable—the plot displays the fitted model parameters with 95% CIs. In Fig. 7G, we performed a partial regression where the residuals from an ordinary least squares regression between “Age” and “GMPD Last 90 Days” were plotted against the residuals from an ordinary least squares regression between “Mean Pseudotime” and “GMPD Last 90 Days”—the former set of residuals served as the independent variable and the latter, as the dependent variable in a regression similar to those in Fig. 7E, I, J.

## Supplementary Material

pgae325_Supplementary_Data

## Data Availability

Whole-blood RNA-seq data for this study have been deposited on the National Center for Biotechnology Information's Gene Expression Omnibus under the accession number: GSE230169. Summarized EpiTOF data are available as supporting data; raw EpiTOF data are accessible on ImmPort under the study accession number: SDY2739.

## Preprints

This manuscript was previously posted as a preprint: https://doi.org/10.1101/2023.04.19.537546.
