# Supplementary material for: Clinical immunity to malaria involves epigenetic reprogramming of innate immune cells
Source: PNAS Nexus. 2024 Aug 6;3(8):pgae325. doi: 10.1093/pnasnexus/pgae325 (PMC11331423; doi:10.1093/pnasnexus/pgae325)
Supplement: pgae325_Supplementary_Data [file pgae325_supplementary_data.docx]

**Supporting Information for**

Clinical Immunity to Malaria Involves Epigenetic Reprogramming of Innate Immune Cells

Jason Nideffer, Maureen Ty, Michele Donato, Rek John, Richard Kajubi, Xuhuai Ji, Felistas Nankya, Kenneth Musinguzi, Kathleen Dantzler Press, Nora Yang, Kylie Camanag, Bryan Greenhouse, Moses Kamya, Margaret E. Feeney, Grant Dorsey, PJ Utz, Bali Pulendran, Purvesh Khatri, Prasanna Jagannathan

Prasanna Jagannathan

Email: [prasj@stanford.edu](mailto:prasj@stanford.edu)

**This PDF file includes:**

Supporting Methods

Figures S1 to S10

Tables S1 to S2

**Supporting Methods**

*Resource Table*

| **Reagent or Resource** | **Source** | **Identifier** |
| --- | --- | --- |
| **EpiTOF Antibodies** | | |
| Monoclonal anti-human CD45, clone HI30 | Thermo Fisher Scientific | (Thermo Fisher Scientific Cat# 14-0459-82, RRID:AB_467274) |
| Monoclonal anti-human H3, clone D1H2 | Cell Signaling Technology | (Cell Signaling Technology Cat# 4499, RRID:AB_10544537) |
| Monoclonal anti-human Arg-me1, clone 5D1 | Abcam | (Abcam Cat# ab415, RRID:AB_304323) |
| Anti-human Arg-me2 (sym) | Cell Signaling Technology | (Cell Signaling Technology Cat# 13222, RRID:AB_2714013) |
| Monoclonal anti-human H3K4me2, clone MABI 0303 | Active Motif | **(Active Motif Cat# 39679, RRID:AB_2793302)** |
| Monoclonal anti-human CD4, clone RPA-T4 | BioLegend | (BioLegend Cat# 300502, RRID:AB_314070) |
| Monoclonal anti-human CD8, clone SK1 | BioLegend | (BioLegend Cat# 344702, RRID:AB_1877104) |
| Monoclonal anti-human H3K9me2, clone 5E5-G5 | BioLegend | (BioLegend Cat# 815501, RRID:AB_2564793) |
| Monoclonal anti-human CD34, clone 8G12 | BD Biosciences | (BD Biosciences Cat# 348050, RRID:AB_400369) |
| Monoclonal anti-human H3K9me1, clone 7E7.H12 | BioLegend | (BioLegend Cat# 824201, RRID:AB_2564878) |
| Monoclonal anti-human H3K36me3, clone RM155 | RevMAb Biosciences | (RevMAb Biosciences Cat# 31-1051-00, RRID:AB_2716375) |
| Monoclonal anti-human H3K27me1, clone MABI 0321 | Active Motif | (Active Motif Cat# 61015, RRID:AB_2715573) |
| Anti-human Arg-me2 (asy) | Cell Signaling Technology | (Cell Signaling Technology Cat# 13522, RRID:AB_2665370) |
| Monoclonal anti-human H3K36me2, clone MABI 0332 | Active Motif | (Active Motif Cat# 61019, RRID:AB_2650523) |
| Monoclonal anti-human H3K27me2, clone MABI 0324 | Active Motif | (Active Motif Cat# 61435, RRID:AB_2793635) |
| Monoclonal anti-human CD11c, clone Bu15 | BioLegend | (BioLegend Cat# 337202, RRID:AB_1236381) |
| Monoclonal anti-human H4K20me2, clone MABI 0422 | Active Motif | (Active Motif Cat# 61533, RRID:AB_2650525) |
| Monoclonal anti-human H3.3, clone EPR17899 | Abcam | (Abcam Cat# ab176840, RRID:AB_2715502) |
| Monoclonal anti-human CD14, clone M5E2 | BioLegend | (BioLegend Cat# 301802, RRID:AB_314184) |
| Monoclonal anti-human H4K20me3, clone 6F8-D9 | BioLegend | (BioLegend Cat# 827701, RRID:AB_2564914) |
| Monoclonal anti-human macro-H2A1.2, clone 14G7 | Millipore | (Millipore Cat# MABE61, RRID:AB_10807977) |
| Monoclonal anti-human H3K4me3, clone G.532.8 | Thermo Fisher Scientific | (Thermo Fisher Scientific Cat# MA5-11199, RRID:AB_10977872) |
| Monoclonal anti-human H2A.Z, clone EPR6171(2)(B) | Abcam | (Abcam Cat# ab150402, RRID:AB_2891240) |
| Monoclonal anti-human H3K36me1, clone EPR16993 | Abcam | (Abcam Cat# ab208581) |
| Monoclonal anti-human H3K27me3, clone MABI 0323 | Active Motif | (Active Motif Cat# 61017, RRID:AB_2614987) |
| Monoclonal anti-human CD33, clone WM53 | BioLegend | (BioLegend Cat# 303402, RRID:AB_314346) |
| Monoclonal anti-human CD16, clone B73.1 | BioLegend | (BioLegend Cat# 360702, RRID:AB_2562693) |
| Monoclonal anti-human H4K20me1 clone 5E10-D8 | Active Motif | (Active Motif Cat# 39727, RRID:AB_2615074) |
| Monoclonal anti-human CD123, clone 9F5 | BD Biosciences | (BD Biosciences Cat# 555642, RRID:AB_395999) |
| Monoclonal anti-human CD3, clone UCHT1 | Biolegend | (BioLegend Cat# 300402, RRID:AB_314056) |
| Monoclonal anti-human CD38, clone HIT2 | BioLegend | (BioLegend Cat# 303502, RRID:AB_314354) |
| Monoclonal anti-human CD56, clone NCAM16.2 | BD Biosciences | (BD Biosciences Cat# 559043, RRID:AB_397180) |
| Monoclonal anti-human H4, clone mAbcam 31830 | Abcam | (Abcam Cat# ab31830, RRID:AB_1209246) |
| Monoclonal anti-human CENP-A, clone 3-19 | MBL International | (MBL International Cat# D115-3, RRID:AB_591074) |
| Monoclonal anti-human CD19, clone HIB19 | BioLegend | (BioLegend Cat# 302202, RRID:AB_314232) |
| Monoclonal anti-human HLA-DR, clone L243 | BioLegend | (BioLegend Cat# 307602, RRID:AB_314680) |
| Monoclonal anti-human γ-H2AX, clone 20E3 | Cell Signaling Technology | (Cell Signaling Technology Cat# 9718, RRID:AB_2118009) |
| Monoclonal anti-human H2BK5ac, clone D5H1S | Cell Signaling Technology | (Cell Signaling Technology Cat# 12799, RRID:AB_2636805) |
| Monoclonal anti-human H3S10ph, clone MABI 0312 | Active Motif | (Active Motif Cat# 39636, RRID:AB_2793285) |
| Monoclonal anti-human H4K5ac, clone MABI0 0405 | Active Motif | (Active Motif Cat# 61523, RRID:AB_2793668) |
| Monoclonal anti-human cleaved H3 (Thr22), clone D7J2K | Cell Signaling Technology | (Cell Signaling Technology Cat# 12576, RRID:AB_2797961) |
| Monoclonal anti-human H3.3S31ph, clone 1A8G10 | Active Motif | (Active Motif Cat# 61671, RRID:AB_2793728) |
| Monoclonal anti-human H3K23ac, clone RM169 | RevMAb Biosciences | (RevMAb Biosciences Cat# 31-1087-00, RRID:AB_2716400) |
| Monoclonal anti-human H3K9ac, clone 2G1F9 | Active Motif | (Active Motif Cat# 61663, RRID:AB_2793725) |
| Monoclonal anti-human H2BS14ph, clone D67H2 | Cell Signaling Technology | (Cell Signaling Technology Cat# 6959, RRID:AB_10891780) |
| Monoclonal anti-human H2AK119ub, clone D27C4 | Cell Signaling Technology | (Cell Signaling Technology Cat# 8240, RRID:AB_10891618) |
| Monoclonal anti-human H3K18ac, clone RM166 | RevMAb Biosciences | (RevMAb Biosciences Cat# 31-1055-00, RRID:AB_2716380) |
| Monoclonal anti-human H3K56ac, clone 12.1 | Active Motif | (Active Motif Cat# 61061, RRID:AB_2793492) |
| Monoclonal anti-human PADI4, clone OTI4H5 | OriGene | (OriGene Cat# TA504813, RRID:AB_2622619) |
| Monoclonal anti-human H2BK120ub, clone D11 | Cell Signaling Technology | (Cell Signaling Technology Cat# 5546, RRID:AB_10693452) |
| Monoclonal anti-human Crotonyl-Lys, clone 4D5 | PTM BIO | (PTM BIO Cat# PTM-502, RRID:AB_2877695) |
| Monoclonal anti-human H3R2cit, clone EPR17703 | Abcam | (Abcam Cat# ab176843) |
| Monoclonal anti-human H3K14ac, clone D4B9 | Cell Signaling Technology | (Cell Signaling Technology Cat# 7627, RRID:AB_10839410) |
| Anti-human H3R2/R8/R17cit | Abcam | (Abcam Cat# ab5103, RRID:AB_304752) |
| Monoclonal anti-human H4K16ac, clone E2B8W | Cell Signaling Technology | (Cell Signaling Technology Cat# 13534, RRID:AB_2687581) |
| Monoclonal anti-human H3K27ac, clone MABI 0309 | Active Motif | (Active Motif Cat# 39685, RRID:AB_2793305) |

*Details of the Clinical Studies*

In all cohorts, upon enrollment all participants were given an insecticide treated bed net and followed for all medical care at a dedicated study clinic. Children who presented with a fever (tympanic temperature >38.0 °C) or history of fever in the previous 24 hours had blood obtained by finger prick for a thick smear. If the thick smear was positive for *Plasmodium* parasites, the patient was diagnosed with malaria regardless of parasite density and treated with artemether-lumefantrine. Symptomatic malaria was defined as any febrile episode accompanied by a positive blood smear requiring treatment. Asymptomatic parasitemia (defined as a positive routine blood smear in the absence of fever) was not treated as per Ugandan ministry of health guidelines.

At select study visits, 3 to 10 milliliters of blood were obtained in EDTA and acid citrate dextrose (ACD) tubes. Blood collected in EDTA tubes (200 μl) was immediately transferred to tubes containing RNA protect. Peripheral blood mononuclear cells (PBMC) were isolated by density gradient centrifugation (Ficoll-Histopaque; GE Life Sciences) from blood collected in ACD tubes, counted, and cryopreserved in liquid nitrogen prior to use.

Samples for RNA Sequencing experiments were obtained from PROMOTE and PRISM2. For RNA Sequencing experiments, paired samples were obtained at two timepoints per subject: when they were uninfected and when they had symptomatic malaria (PROMOTE), or when they were uninfected and when they had asymptomatic parasitemia as detected by qPCR (PRISM2).

Samples for EpiTOF were obtained from PRISM1. For EpiTOF experiments, paired samples were obtained at three timepoints per subject: when they were uninfected, when they had symptomatic malaria, or when they had asymptomatic parasitemia. The ordering of sampling with respect to disease state differed between individuals to ensure that age and time of sampling would not confound comparisons across disease states.

*Epigenetic landscape profiling using cytometry by time of flight (EpiTOF)*

Cryopreserved PBMCs were shipped to Stanford University, thawed and incubated in RPMI 1640 media (ThermoFisher) containing 10% FBS (ATCC) at 37°C for 1 hour prior to processing. Cisplatin (ENZO Life Sciences) was added to 10 mM final concentration for viability staining for 5 minutes before quenching with CyTOF Buffer (PBS (ThermoFisher) with 1% BSA (Sigma), 2mM EDTA (Fisher), 0.05% sodium azide). Cells were centrifuged at 400 g for 8 minutes and stained with lanthanide-labeled antibodies against immunophenotypic markers in CyTOF buffer containing Fc receptor blocker (BioLegend) for 30 minutes at room temperature (RT). Following extracellular marker staining, cells were washed 3 times with CyTOF buffer and fixed in 1.6% PFA (Electron Microscopy Sciences) at 1x106 cells/ml for 15 minutes at RT. Cells were centrifuged at 600 g for 5 minutes post-fixation and permeabilized with 1 mL ice-cold methanol (Fisher Scientific) for 20 minutes at 4C. 4 mL of CyTOF buffer was added to stop permeabilization followed by 2 PBS washes. Mass-tag sample barcoding was performed following the manufacturer’s protocol (Fluidigm). Individual samples were then combined and stained with intracellular antibodies in CyTOF buffer containing Fc receptor blocker (BioLegend) overnight at 4C. The following day, cells were washed twice in CyTOF buffer and stained with 250 nM 191/193Ir DNA intercalator (Fluidigm) in PBS with 1.6% PFA for 30 minutes at RT. Cells were washed twice with CyTOF buffer and once with double-deionized water (ddH2O) (ThermoFisher) followed by filtering through 35 mm strainer to remove aggregates. Cells were resuspended in ddH2O containing four element calibration beads (Fluidigm) and analyzed on CyTOF2 (Fluidigm).

*Statistical Analyses of EpiTOF Cluster Frequencies*

Fisher’s exact test (two-tailed) was used to compare the frequency of each meta cluster across disease states (as in Figure 5D). Specifically, a contingency table was made for each comparison where, on one axis, cells were counted based on whether they were “inside” or “outside” of a given cluster and, on the other axis, cells were counted whether they were a part of one disease state or another (depending on the pairwise comparison). P-values generated from the tests performed relative to each cluster were adjusted using the Benjamini-Hochberg procedure. These tests were prespecified, while follow-up statistical tests specifically examining clusters C and G were specified post-hoc. In cases where meta cluster frequencies were calculated on a per-sample basis (as in Figure 5E-G), paired two-tailed t-tests were used to compare these frequencies between the symptomatic and asymptomatic disease states. The relative abundance of a meta cluster within a specific immune cell population in children with symptomatic malaria versus asymptomatic parasitemia was calculated using Hedges’ g formula (as in Figure 5G). Regressions involving clinical variables were frequently illustrated as linear relationships with lines of best fit and 95% confidence intervals. For these relationships, Spearman correlations were also performed with rho values and associated p values displayed.

*Calculating Clinical Variables*

Clinical variables were calculated as follows: “Malaria Incidence Future” = (# of malaria episodes over the next three years) / 3 years; “Test-Positive Rate Future” = (# of positive blood smears performed over the next three years) / (# of total blood smears performed over the next three years). “Malaria Incidence Past” and “Test-Positive Rate Past” were calculated similarly; however, these clinical variables utilized available data from prior to sampling. “log10(GMPD)” = log-base-10 transformation of the geometric mean of all (parasite density values + 1) measured over the next three years; “NMF Incidence Future” = (# of non-malarial fevers observed over the next three years) / 3 years; “Future Risk of Malaria if Parasitemic” = Malaria Incidence Future / (Test-Positive Rate Future x 365); “Past Risk of Malaria if Parasitemic” = Malaria Incidence Past / (Test-Positive Rate Past x 365).

***
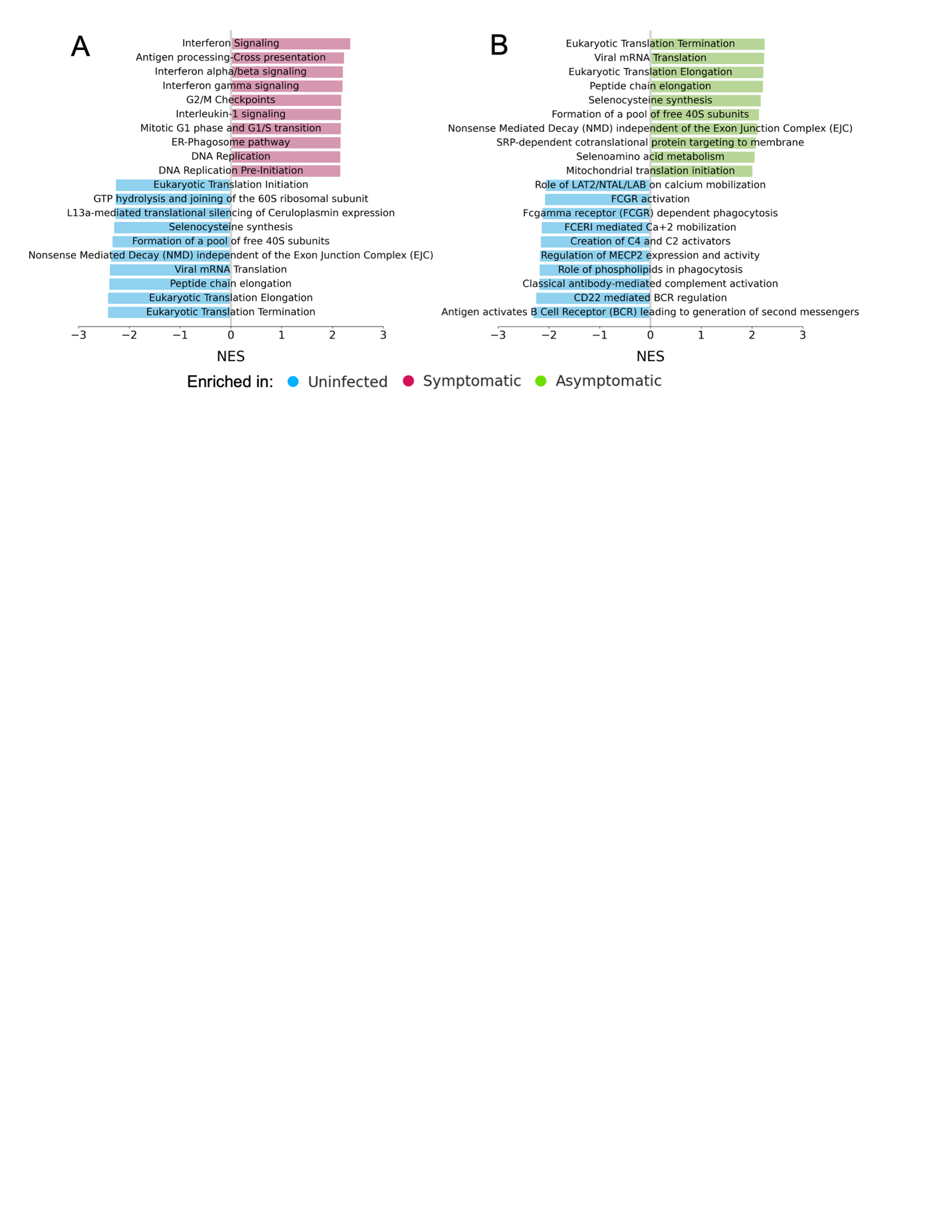
 Figure S1****. Gene set enrichment analysis of whole-blood transcriptomic data assessing the active pathways in symptomatic and asymptomatic parasitemia compared to an uninfected baseline.* (A-B) Normalized enrichment scores (NES) for ten most enriched and ten most depleted Reactome gene sets in (A) symptomatic versus uninfected and (B) asymptomatic versus uninfected.

***
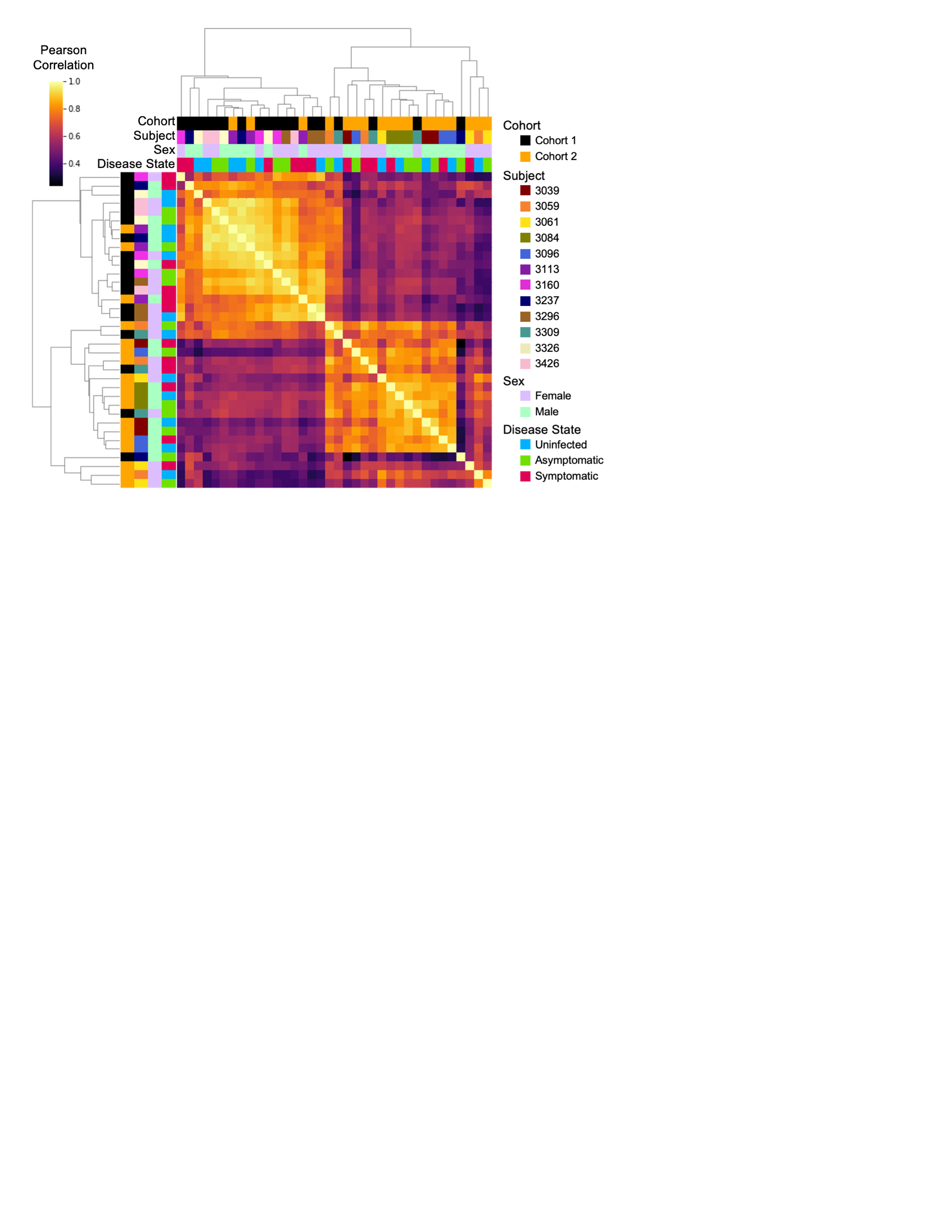
***

***Figure S2****. Correlation heatmap describing the inter- and intra-cohort heterogeneity of samples analyzed by EpiTOF.* Variables used for correlations included average normalized expression of each of the 40 epigenetic markers within each of the 11 annotated cell populations (440 variables total).

***Figure S3****. Gating strategy for identifying surface marker defined cell populations from EpiTOF experiments.* (A) Gating of annotated cell populations distinct from NK cells. (B) Gating of NK cells.

***Figure S4****. Forest plots showing EpiTOF markers differentially expressed in children when they had asymptomatic parasitemia versus when they were uninfected.* 95% confidence intervals are shown for each cohort separately and for the summarized effect size. All displayed markers yielded a p-value less than 0.01.

***Figure S5****. Forest plots showing EpiTOF markers differentially expressed in children when they had symptomatic malaria versus when they were uninfected.* 95% confidence intervals are shown for each cohort separately and for the summarized effect size. All displayed markers yielded a p-value less than 0.01.

***Figure S6****. Forest plots showing EpiTOF markers differentially expressed in children when they had symptomatic malaria versus when they had asymptomatic parasitemia.* 95% confidence intervals are shown for each cohort separately and for the summarized effect size. All displayed markers yielded a p-value less than 0.01.

***Figure S7****. Gating strategy for monocytes stimulated and analyzed by flow cytometry.* (A) Gating of classical and CD16+ monocytes. (B) Stimulation with Pam3CSK4 kills CD16+ monocytes but not classical monocytes. (C-D) Representative flow plots showing TNFα and IL-6 expression of classical monocytes after no stimulation (C) or Pam3CSK4 stimulation (D).

***
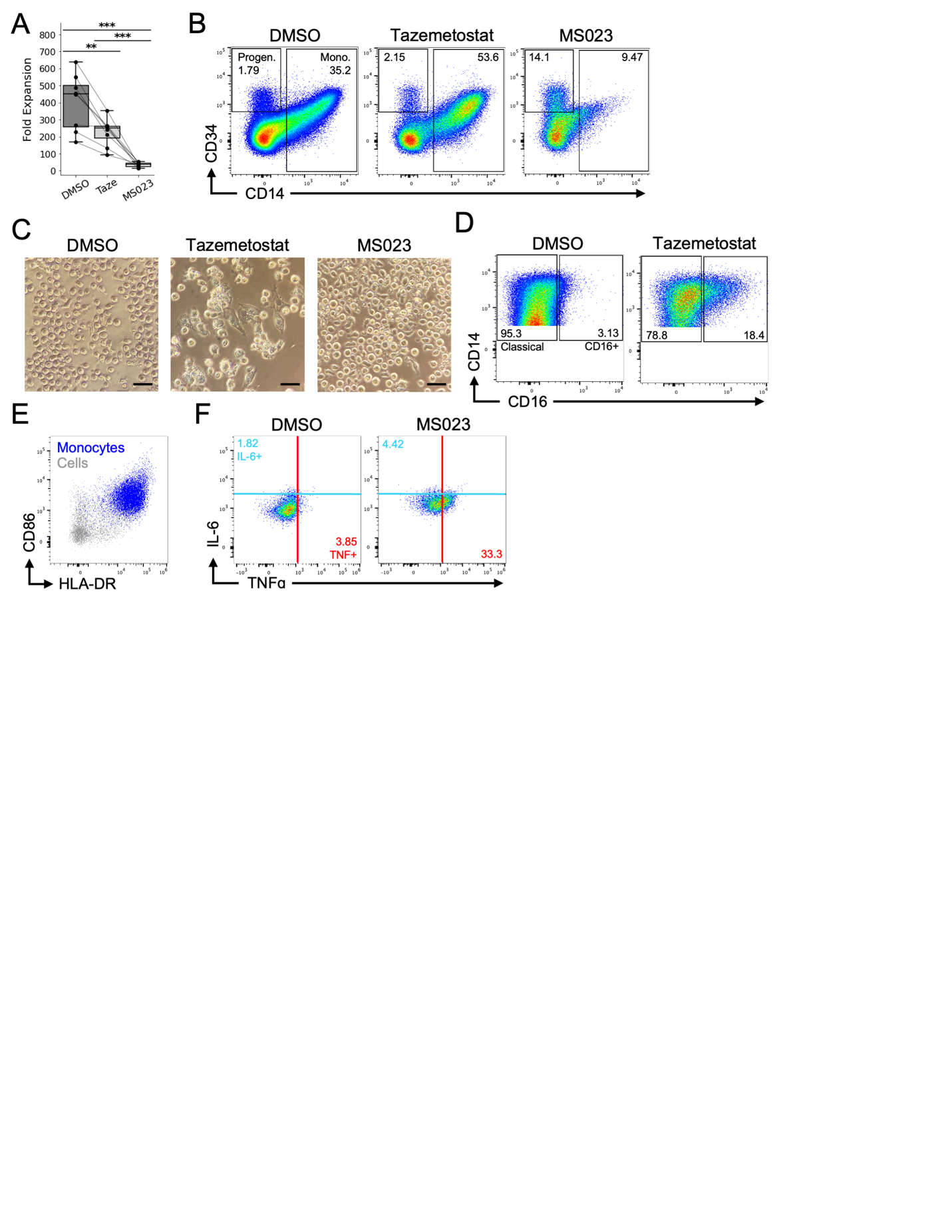
***

***Figure S8****. Expansion and phenotype of monocytes differentiated from cord blood progenitors in the presence or absence of methyltransferase inhibitors.* (A) Fold expansion of cells on culture day 12. Statistical significance was determined by performing pairwise, paired T tests. p-values were corrected using the Benjamini-Hochberg procedure. p-value < 0.05 (*); p-value < 0.01 (**); p-value < 0.001 (***). (B) Gating of CD34+ progenitors and CD14+ monocytes on day 12 of differentiation in the presence of either DMSO, tazemetostat, or MS023. (C) Light microscopy images taken on day 12 of cells cultured in the presence of methyltransferase inhibitors. Scale bar represents 50 microns. (D) Gating of classical (CD16-) and CD16+ monocytes. (E) Representative example of HLA-DR and CD86 expression by monocytes and other cells on day 12. (F) Gating of TNFα and IL-6 expressing progenitors on day 12.

***
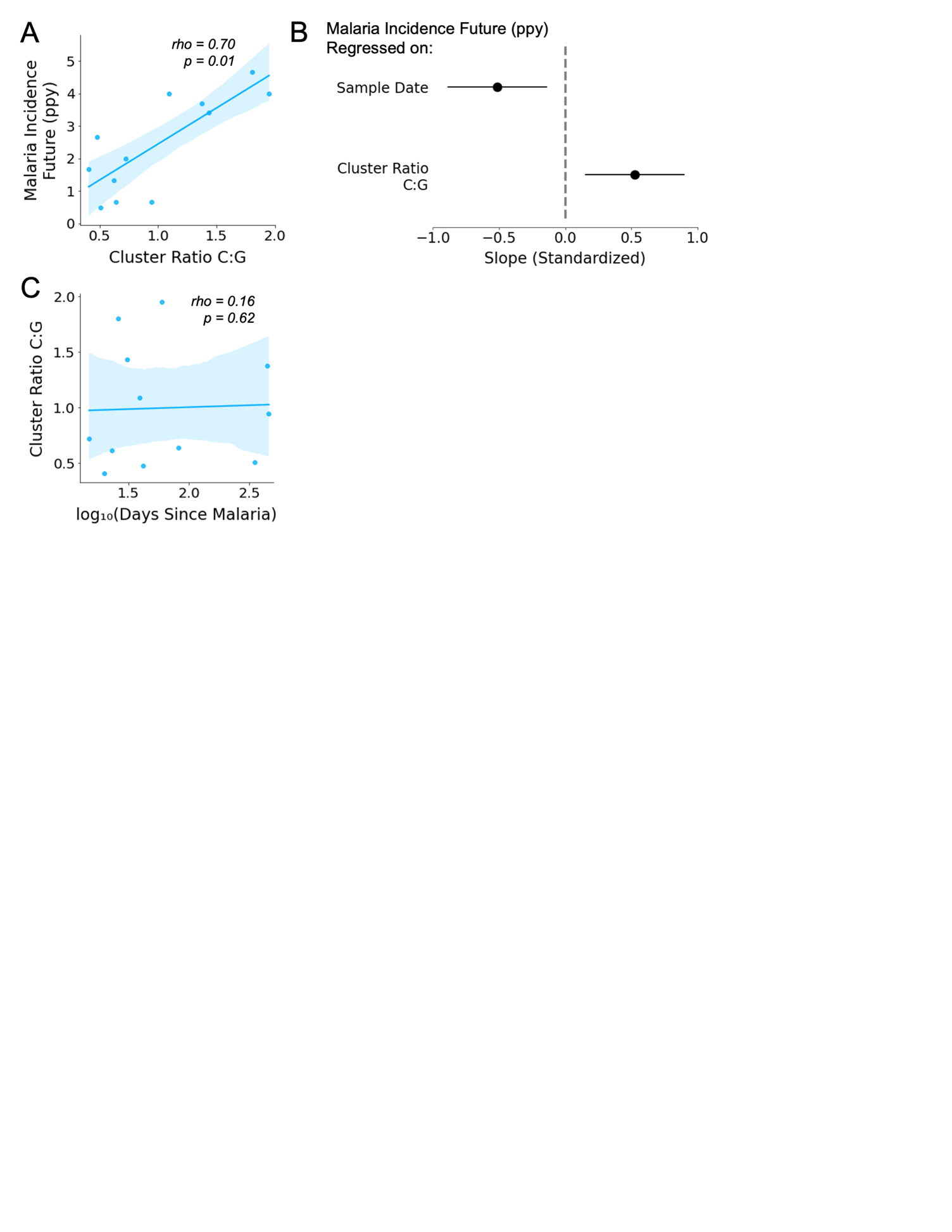
***

***Figure S9****. Association between epigenetics and future incidence of malaria.* (A) Scatter plot depicting the relationship between the ratio of cluster C to cluster G (C:G) and future incidence of malaria. (B) Fitted parameter values from a multiple regression where the future incidence of malaria was regressed on sample date and cluster ratio (C:G)—values of fitted parameters are shown with 95% confidence intervals. Independent and dependent variables were standardized prior to model fitting. (C) Scatter plot depicting the relationship between the days since symptomatic malaria (log-base-10 transformed) and the ratio of cluster C to cluster G (C:G) and future incidence of malaria. For A and ‘C’, the lines and shaded regions depict fitted linear least squares regressions with 95% confidence intervals. Rho and p-values are reported for Spearman correlations.


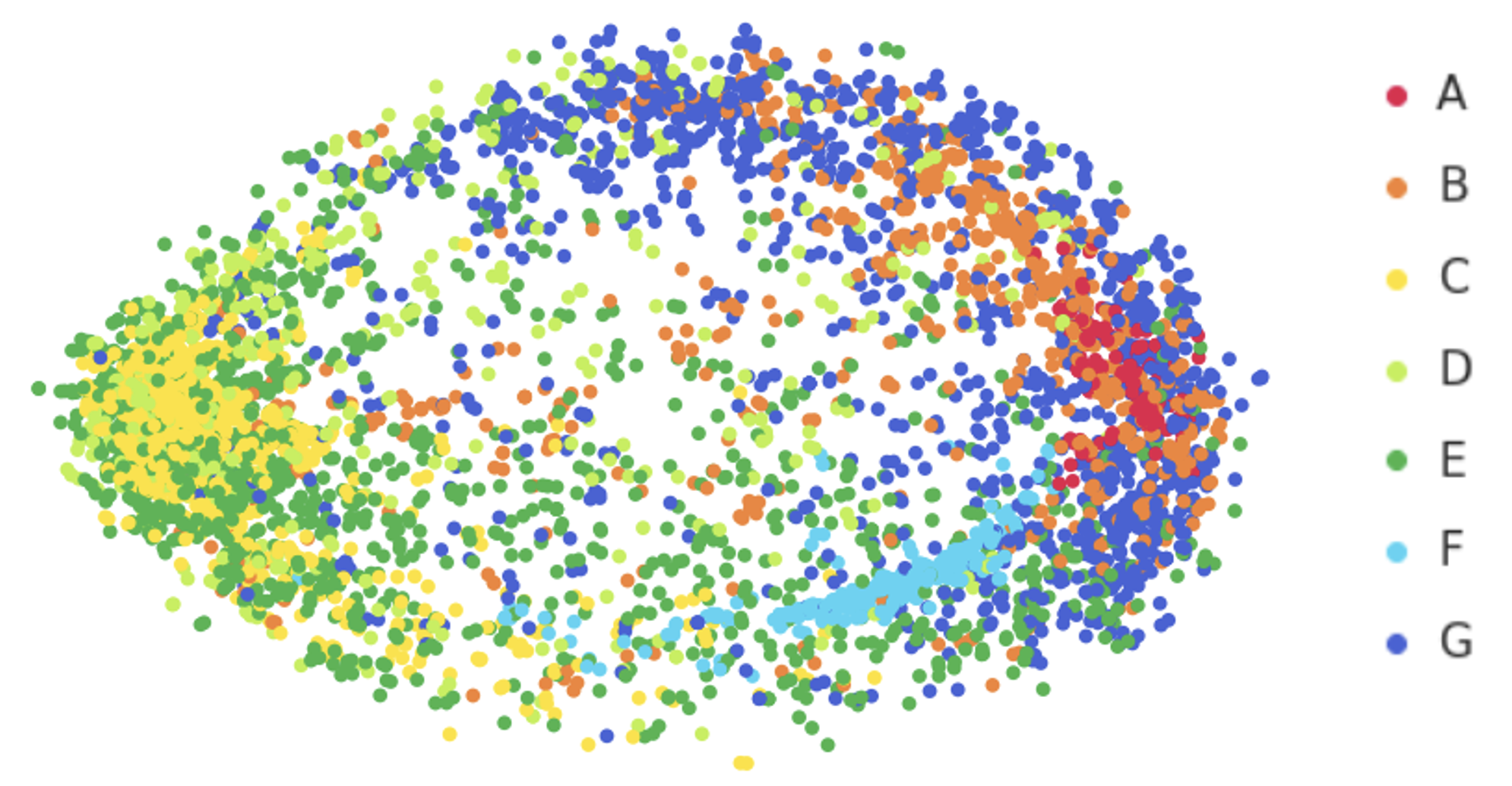


***Figure S10****. Epigenetically-defined populations projected in trajectory space.* tSpace projection identical to the one in Figure 7. Cells are colored according to their epigenetically-defined cluster identities.

***Table S1****.* Demographics and other characteristics of cohorts analyzed by whole-blood RNA sequencing.

| RNA Sequencing Cohorts | | |
| --- | --- | --- |
| Symptomatic malaria cohort (n=17, paired) |  |  |
|  | **Uninfected** | **Symptomatic** |
| Age in years, mean (SD) | 0.6 (0.11) | 0.6 (0.10) |
| Male gender, n (%) | 10 (58.8%) | 10 (58.8%) |
| Parasite density by blood smear, p/μl (range) | 0 | 39,000 (112-177000) |
| Sickle cell trait | 3 (17.7%) | 3 (17.7%) |
| Asymptomatic parasitemia cohort (n=14, paired) |  |  |
|  | **Uninfected** | **Asymptomatic** |
| Age in years, mean (SD) | 5.8 (2.8) | 5.9 (2.8) |
| Male gender, n (%) | 8 (57%) | 8 (57%) |
| Parasite density by qPCR, p/μl (range) | 0 | 918 (0.1-9035) |
| Blood smear positive individuals, n (%) | 0 | 4 (28.6%) |
| Sickle cell trait | 6 (42.8.0%) | 6 (42.8%) |

***Table S2*.** Demographics and other characteristics of cohorts analyzed by EpiTOF.

| EpiTOF Cohorts | | | |
| --- | --- | --- | --- |
| Cohort 1 (n=6, paired) |  |  |  |
|  | **Uninfected** | **Symptomatic** | **Asymptomatic** |
| Age in years, mean (SD) | 6.24 (0.78) | 6.64 (0.67) | 6.37 (1.03) |
| Male gender, n (%) | 3 (50%) | 3 (50%) | 3 (50%) |
| Parasite density by blood smear, p/μl (range) | 0 | 29000  (8160-56200) | 4539  (272-20000) |
| Sickle cell trait | 2 (33.3%) | 2 (33.3%) | 2 (33.3%) |
| Cohort 2 (n=6, paired) |  |  |  |
|  | **Uninfected** | **Symptomatic** | **Asymptomatic** |
| Age in years, mean (SD) | 6.67 (1.28) | 6.78 (1.50) | 6.50 (1.18) |
| Male gender, n (%) | 3 (50%) | 3 (50%) | 3 (50%) |
| Parasite density by blood smear, p/μl (range) | 0 | 9182  (336-30840) | 7787  (80-38880) |
| Sickle cell trait | 2 (33.3%) | 2 (33.3%) | 2 (33.3%) |
